# Epitope-specific competitive ELISA predicts malaria transmission-blocking vaccine Pfs230D1 activity measured in standard membrane feeding assay

**DOI:** 10.1172/jci.insight.198414

**Published:** 2026-01-01

**Authors:** Cristina A. Meehan, Matthew V. Cowles, Robert D. Morrison, Yuyan Yi, Jingwen Gu, Jen C.C. Hume, Mina P. Peyton, Issaka Sagara, Sara A. Healy, Jonathan P. Renn, Patrick E. Duffy

**Affiliations:** 1 Laboratory of Malaria Immunology and Vaccinology, Division of Intramural Research, National Institute of Allergy and Infectious Diseases, NIH, Bethesda, Maryland, USA.; 2 Medical Scientist Training Program, Heersink School of Medicine, University of Alabama at Birmingham, Birmingham, Alabama, USA.; 3 Bioinformatics and Computational Biosciences Branch, Office of Cyber Infrastructure and Computational Biology, National Institute of Allergy and Infectious Diseases, NIH, Bethesda, Maryland, USA.; 4 Malaria Research and Training Center, University of Sciences, Techniques and Technologies, Bamako, Mali.

**Keywords:** Immunology, Infectious disease, Immunoglobulins, Malaria, Vaccines

## Abstract

Functional antibody responses to malaria transmission-blocking vaccines (TBVs) are assessed using the standard membrane feeding assay (SMFA). This assay quantifies the percentage reduction of oocyst levels in mosquitoes fed gametocytes mixed with antisera/antibodies, referred to as transmission-reducing activity (TRA). As TBVs advance to large clinical trials, new scalable assays are needed to characterize vaccine responses. Here, we developed an epitope-specific competitive ELISA platform (P230Compete) for TBV candidate Pfs230D1, based on single-chain variable fragments against epitopes recognized by human monoclonal antibodies with high TRA. We quantified functional epitope-specific antibody responses (F) in phase I Pfs230D1-EPA/AS01 vaccine trial participants, using 171 serum samples collected at 2 postvaccination time points. Five antibody features were examined by P230Compete, including total IgG (reported as ELISA units, EU_F_), IgG subclasses (IgG1_F_, IgG3_F_, IgG4_F_), and bound complement factor C1q (C1q_F_). EU_F_ and IgG1_F_ demonstrated strong correlation and excellent prediction of TRA (≥80%) in logistic regression analysis (AUC of 0.81 for both assays after dose 3, and 0.80 and 0.76 after dose 4). Furthermore, combining EU_F_ and IgG1_F_ showed even better predictive performance at each time point. P230Compete offers a promising proxy assay to replace SMFA in late-stage Pfs230D1 trials.

## Abstract 2

A novel epitope-specific competitive ELISA for leading malaria transmission-blocking vaccine Pfs230D1 predicts serum activity to reduce mosquito infections measured in standard membrane feeding assays.

## Introduction

Malaria remains a global health threat, with an estimated 597,000 deaths and 263 million infections worldwide in 2023 (1). Drug-resistant parasites, insecticide-resistant mosquitoes, and the spread of urban vector *Anopheles stephensi* across Africa jeopardize recent gains in malaria control, underscoring the need for novel interventions (2, 3). RTS,S and R21, the first licensed malaria vaccines (2, 4) are pre-erythrocytic vaccines that target sporozoite stages of *Plasmodium falciparum* and are approved for the prevention of clinical malaria in young children (5–8). Alternatively, malaria transmission-blocking vaccines (TBVs) induce antibodies that kill parasites in the mosquito midgut to block transmission and pursue malaria elimination by herd immunity (9–11). Pfs230 domain 1 (Pfs230D1), a recombinant fragment of *P*. *falciparum* gamete surface antigen Pfs230, is the most advanced TBV candidate and has completed phase I and phase II field trials in Mali (12–14). Vaccination with Pfs230D1 induces complement-dependent functional antibodies that lyse gametes in the mosquito midgut, thereby interrupting transmission (14–16).

Historically, TBV-induced serum activity has been assessed by the standard membrane feeding assay (SMFA) (17, 18), a mosquito-based in vitro assay employed in both preclinical and early-stage clinical studies (19–21). In SMFA, insectary-raised *Anopheles* mosquitoes feed through a membrane on cultured *P*. *falciparum* gametocyte-infected erythrocytes, mixed with either control or immune sera/IgG. One week later, mosquitoes are dissected and midgut oocysts counted; transmission-reducing activity (TRA) is calculated as the percentage reduction in oocyst numbers in the presence of immune versus control sera or IgG (17, 22). TRA of 80% or higher is generally considered to be a high and reproducible level of activity measured in SMFA (17, 23). The range of reliable TRA quantification is limited: TRA from 50% to 80% can vary between assays, and values below 50% are often considered indeterminate or negative (23, 24).

SMFA remains a valuable tool but has limitations: it is labor intensive, time-consuming, and low throughput (20, 24, 25). Dependence on parasite culture, complex biosafety level insectaries, and colony-raised mosquitoes pose a challenge for scalability in low-resource settings (20). Factors like temperature, humidity, mosquito fitness, and microbiota can introduce variability in oocyst levels (26). The commonly used *P*. *falciparum* NF54 strain, maintained under laboratory conditions for decades, generates high gametocyte numbers but does not reflect the biological or genetic diversity of field isolates (17, 21). These limitations underscore the need for a practical, scalable, in vitro surrogate for mosquito-based assays to support the large number of samples required during late-stage TBV clinical development (12, 14, 27).

ELISA, in contrast, offers standardized, same-day, reproducible results. Epitope-specific competitive ELISA (ESC-ELISA) measures targeted responses to protective epitopes, rather than total antigen-specific IgG; a critical distinction, as only a subset of vaccine-induced antibodies mediates protective functions (15, 16, 27). ESC-ELISAs have been developed in the context of several infectious diseases, including dengue, HIV, SARS-CoV-2, influenza, and malaria (28–32). ESC-ELISA has been used to measure epitope-specific responses for diagnosis and measurement of functional antibody responses.

To address the need for a proxy to SMFA, we developed an ESC-ELISA platform, hereafter referred to as P230Compete. P230Compete targets functional epitopes of Pfs230D1 (15, 16) to estimate functional antibody in serum. P230Compete was adapted to measure 4 antibody classes or subclasses (total IgG, IgG1, IgG3, IgG4) as well as complement binding (C1q). A pilot study using 171 serum samples from a Pfs230D1 vaccine trial allowed us to evaluate P230Compete levels as a correlate of high serum functional activity (defined as TRA ≥ 80%) measured by SMFA. The assay results were both correlative to and predictive of TRA, demonstrating the potential of P230Compete as a scalable, low-cost proxy to SMFA, suitable for use in late-stage TBV trials.

## Results

### P230Compete measures serum reactivity to functional epitopes.

**Figure 1:**
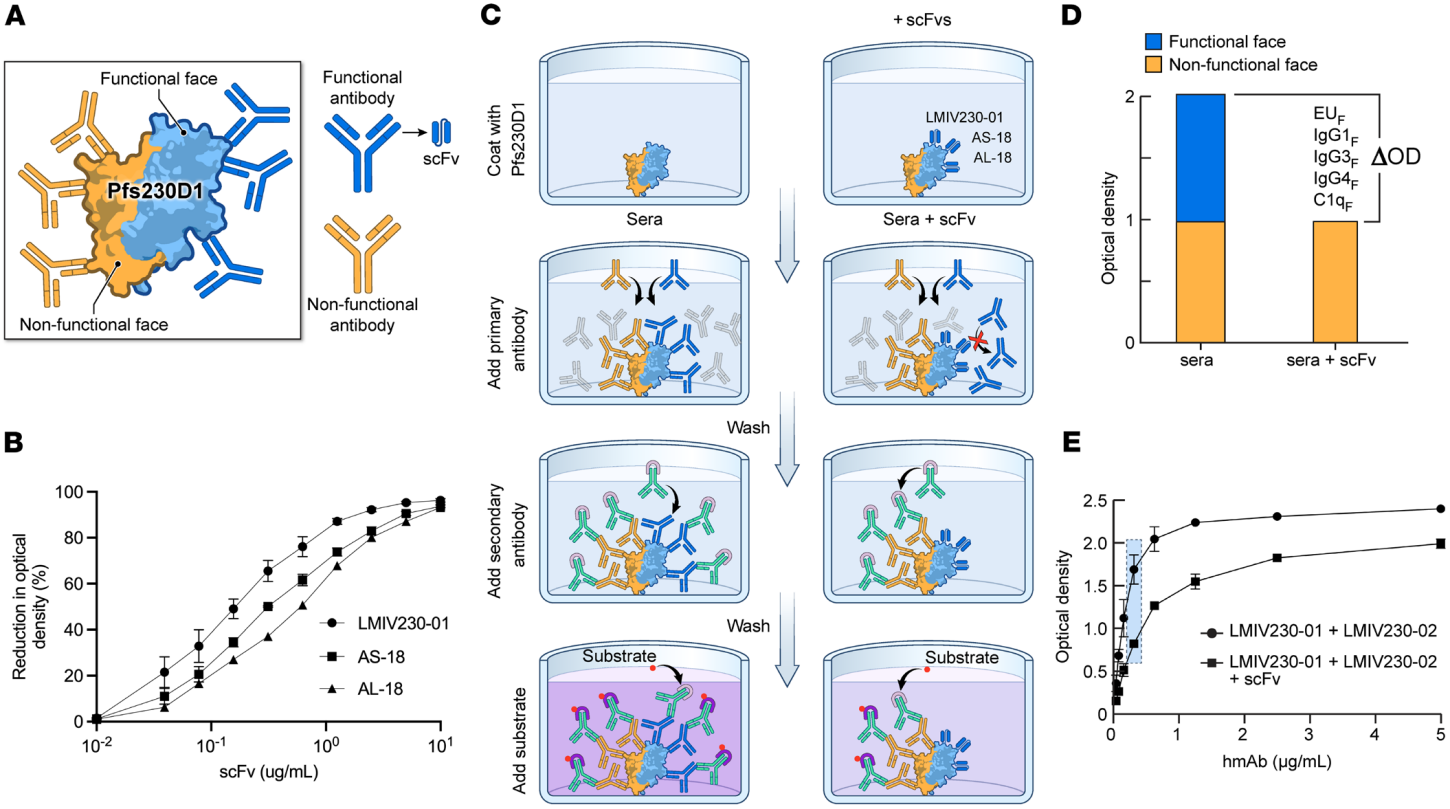
P230Compete platform measures antibody displacement from Pfs230D1 functional epitopes. (**A**) Functional (blue) and nonfunctional (gold) faces of Pfs230D1 depicted. Functional hmAbs (blue) bound to 3 distinct epitope bins on the functional face; nonfunctional hmAbs (gold) bound to the opposing face. (**B**) scFv-mediated displacement of functional hmAbs (LMIV230-01, 230AS-18, and 230AL-18), each at 0.1 mg/mL by corresponding scFv (scFv_LMIV230-01,_ scFv_230AS-18_, scFv_230AL-18_), and each at a saturating concentration of 10 mg/mL. A greater than 90% reduction in OD was demonstrated for all 3 experiments (*n* = 5 LMIV230-01; *n* = 3 AS-18; *n* = 2 AL-18); data shown as mean ± SD. (**C**) P230Compete protocol: wells coated with 0.1 mg/mL Pfs230D1; after blocking, trial participant sera added with or without scFv; after washing, labeled secondary antibody added to quantify bound IgG or IgG features. Two OD levels were measured: OD_Sera_ in the absence of scFv (dark purple) and OD_Sera+scFv_ measured in the presence of scFv (light purple). (**D**) ΔOD = OD_Sera_ – OD_Sera+scFv_ and reported as IgG1_F_, IgG3_F_, IgG4_F_, and C1q_F_, or ΔEU = EU_Sera_ – EU_Sera+scFv_ and reported as EU_F_. “F” subscript denotes displacement of serum antibody from the Pfs230D1 functional face (blue). (**E**) A 1:1 mixture of functional (LMIV230-01) and nonfunctional (LMIV230-02) hmAbs was tested alone (circles) or in combination with 10 μg/mL saturating scFv_LMIV230-01_ (squares) (*n* = 3). At 0.375 μg/mL total hmAb concentration (blue box), OD decreased from 1.6 (circle, no scFv) to 0.8 (square, with scFv), indicating a ΔOD of 0.8 by competitive inhibition of scFv; data shown as mean ± SD.

Recent structural studies of Pfs230D1 bound to human mAb (hmAb) have defined 2 distinct antigen faces (Figure 1) (15). One face (referred to hereafter as the functional face) reacts to hmAb with high TRA, and an opposing nonfunctional face reacts to low TRA hmAb (15, 16), likely due to epitope occlusion by downstream domains in the native antigen. P230Compete targets the Pfs230D1 functional face with single-chain variable fragments (scFvs) generated from Pfs230D1 vaccinees (14) to competitively bind functional epitopes and indirectly measure serum functional antibody responses through their displacement.

Epitope-mapping studies defined 3 distinct bins on the functional face of Pfs230D1 (Figure 1A), including a bin with functional hmAbs LMIV230-01 and 230AS-73, a bin with 230AS-18 and 230AL-37, and a bin with 230AL-18 (15). To block the functional face in the P230Compete platform, we prepared scFvs based on 3 nonoverlapping epitopes: scFv_LMIV230-01_, scFv_230AL-18_, and scFv_230AS-18_. In optimization experiments, we confirmed epitope-specific competition with each scFv displacing more than 90% of hmAb binding at 10 mg/mL of scFv: 96.5% for scFv_LMIV230-01_, 93.2% for scFv_230AL-18_, and 95.4% for scFv_230AS-18_ (Figure 1B). No further displacement was observed beyond 10 mg/mL of scFv, identifying the saturating concentration required to fully block the Pfs230D1 functional face. Serum antibody displacement was quantified as the difference between 2 OD values: OD measured in the presence of scFv (hereafter referred to as OD_Sera+scFv_) or absence of scFv (OD_Sera_) (Figure 1C). The difference in OD (or ELISA units, EU) was referred to as ΔOD (or ΔEU) and was the primary outcome of interest for the P230Compete assay panel: EU_F_ (total IgG, reported as ΔEU), IgG1_F_, IgG3_F_, IgG4_F_, and C1q_F_ (all reported as ΔOD), where subscript “F” denotes functional antibody displacement (Figure 1D).

To first assess the assay platform, a mixture of LMIV230-01 (functional hmAb) and LMIV230-02 (nonfunctional hmAb) was prepared at approximately 1:1 ratio and analyzed by P230Compete. In the presence of scFv_LMIV230-01_, reactivity of the hmAb mixture to Pfs230D1 decreased from 1.6 OD to 0.8 OD (Figure 1E), demonstrating a ΔOD of 0.8. The 50% reduction in OD was consistent with selective hmAb displacement of LMIV230-01. The effect of serum matrix on scFv-mediated competition (Supplemental Figure 1; supplemental material available online with this article; https://doi.org/10.1172/jci.insight.198414DS1) was evaluated by performing the assay with or without 1:500 diluted malaria-naive serum; ΔOD values were similar under both conditions, confirming no interference by serum matrix for scFv-mediated competition.

### P230Compete pilot study of sera from a Pfs230D1 vaccine trial.

**Figure 2:**
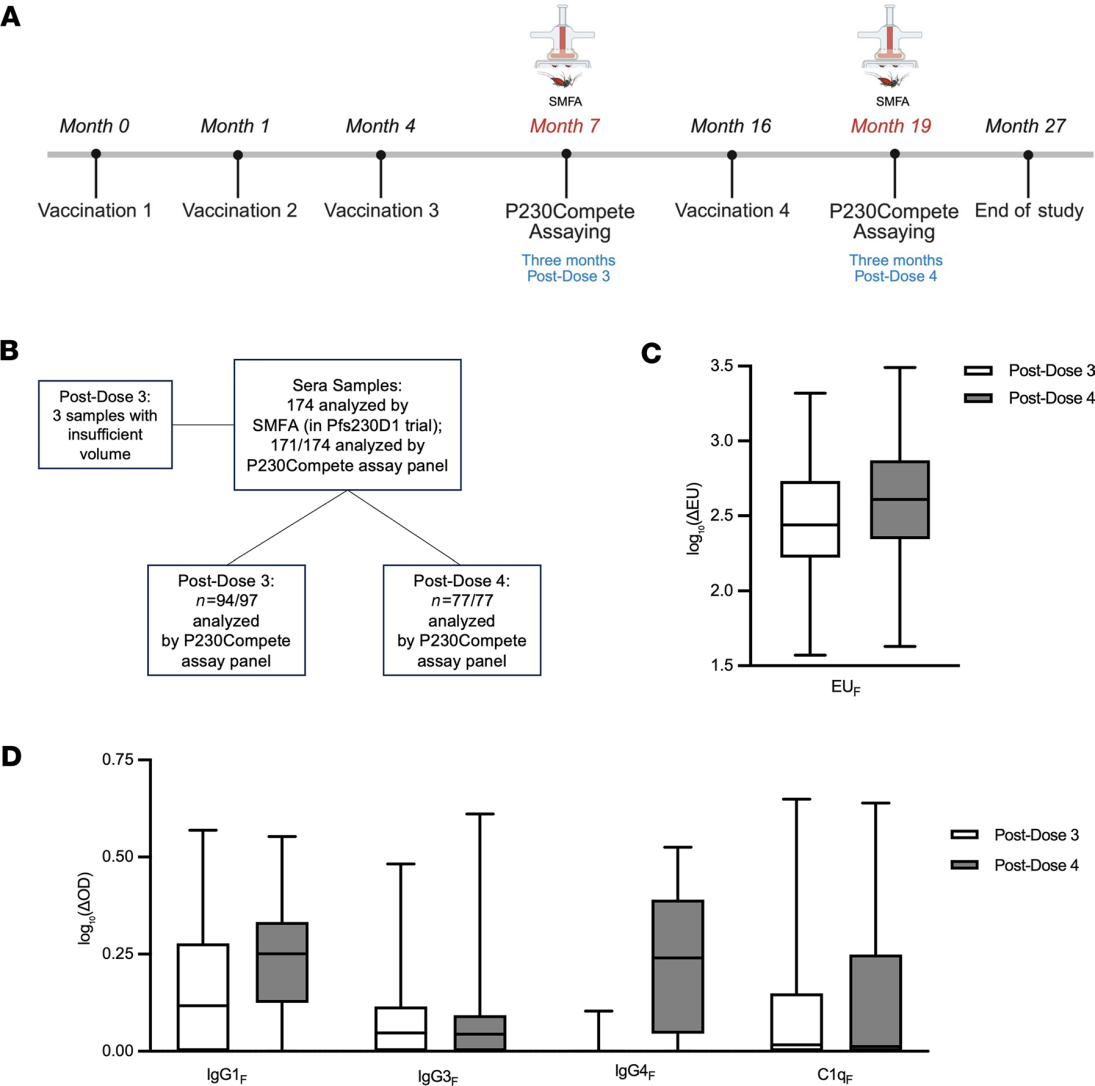
P230Compete pilot study of human serum from Pfs230D1 trial. (**A**) Samples for this study were collected at month 7 (3 months after dose 3) and month 19 (3 months after dose 4), corresponding to time points at which the standard membrane feeding assay (SMFA) was performed during the trial. (**B**) 171/174 samples analyzed by SMFA during the Pfs230D1 vaccine trial were analyzed by the P230Compete assay panel (EU_F_, IgG1_F_, IgG3_F_, IgG4_F_, and C1q_F_) for the pilot study reported here. (**C**) Box-and-whisker plots show the distribution of data for total IgG P230Compete results (referred to as EU_F_, expressed as log-transformed ELISA units: EU) after dose 3 (clear) and after dose 4 (gray); the center line represents the median, the box edges show the 25th and 75th percentiles (IQR), and the whiskers extend to the most extreme points within 1.5 × IQR. (**D**) Levels of IgG subclasses (IgG1_F_, IgG3_F_, and IgG4_F_) and C1q binding (C1q_F_) with “F” denoting displacement of antibody from the functional face of Pfs230D after dose 3 (clear) and after dose 4 (gray).

After developing the P230Compete platform, we implemented the assay panel in a pilot study using sera from a phase I trial of Pfs2301-EPA/AS01 in Malian adults (14). Participants in the trial were randomized 1:1:2 to 3 arms: Pfs230D1 full dose, Pfs230D1 fractional dose, and a comparator vaccine (ENGERIX-B/Menactra), and received 4 vaccine doses at months 0, 1, 4, and 16 (Figure 2). Sera collected at 3 months after dose 3 and 3 months after dose 4 of Pfs230D1 were selected for P230Compete assay panel analysis (Figure 2A). As sera from these time points were also assayed in SMFA during the trial, the P230Compete assay results could be correlated to TRA and assessed for prediction of TRA 80% or higher (Supplemental Table 1 and Supplemental Figure 2).

A total of 174 serum samples were assayed in SMFA during the trial from both the full- and fractional-dose cohorts as reported previously (14). Of these, 171 had sufficient volume for analysis by the P230Compete assay panel (Figure 2B): the 3 missing samples were drawn from 2 participants with samples of insufficient volume after dose 3 but samples available after dose 4, and 1 participant with sample of insufficient volume after dose 3 (and with no sample collected after dose 4). For TRA prediction analyses presented here, results from vaccinees who received full- or fractional-dose regimens were combined after dose 3 and after dose 4 because neither baseline demographics nor TRA levels differed significantly between these dosing groups, as described previously (14).

The total number of samples analyzed by the P230Compete assay panel included 94 samples after dose 3 and 77 samples after dose 4. Because of limited sample volumes, the C1q_F_ assay was performed on only 87 of 94 post-dose 3 samples and 60 of 77 post-dose 4 samples (see Methods). Additionally, 1 sample after dose 3 was unavailable for IgG3_F_ analysis, resulting in a total of 93 samples analyzed for that dataset. Six samples from participants in the comparator arm, who did not receive the Pfs230D1 vaccine, were included in the analysis as a negative control for both post-dose 3 and post-dose 4 samples; OD_Sera_ levels for the comparator samples were below the limit of detection (LOD), and thus their P230Compete results were not included in the TRA prediction analyses (see Methods).

All available post-dose 3 and post-dose 4 samples were analyzed across the P230Compete assay panel. P230Compete EU_Sera_ and OD_Sera_ levels (Table 1 and Supplemental Figure 3) were compared with EU and OD values measured by direct ELISA during the trial, as reported previously (14). Despite the 10-fold reduction in Pfs230D1 antigen concentration used to adsorb onto P230Compete plates, EU_Sera_ and OD_Sera_ values correlated significantly (Supplemental Figure 4) with the corresponding total IgG and subclass-specific Pfs230D1 titers measured during the trial (14). EU_Sera_ levels for total IgG demonstrated strong correlation with total IgG Pfs230D1 titers measured during the trial (hereafter referred to as EU_TOTAL_) after dose 3 (ρ = 0.82) and after dose 4 (ρ = 0.74, both *P* < 0.0001). OD_Sera_ levels for IgG subclasses (IgG1, IgG3, and IgG4) after both vaccinations correlated moderately with IgG subclass levels measured during the trial (ρ = 0.46–0.67; all *P* < 0.0001).

P230Compete results for ΔEU and ΔOD were generally higher after dose 4 in comparison to dose 3 (Figure 2, C–D, and Table 1). EU_Sera_ responses were detectable in 100% of participants at both time points (94 of 94 after dose 3; 77 of 77 after dose 4), with the mean ΔEU increasing from 410 ± 371 (range: 36–2,068) to 592 ± 572 (range: 42–3,081) (Figure 2C). IgG1 responses were also detectable in most samples at both time points, with 63 of 94 samples (67%) above LOD after dose 3 and 68 of 77 (88%) after dose 4. Corresponding mean ΔOD values increased from 0.563 ± 0.665 (range: 0.00–2.71) to 0.763 ± 0.549 (range: 0.00–2.57) (Figure 2D), indicating an increased and more uniform IgG1 signal after the booster dose.

IgG3 responses showed limited changes across time points, with 65 of 93 (70%) and 56 of 77 (73%) samples above LOD after dose 3 and after dose 4, respectively. Mean ΔOD values for IgG3 remained modest (mean ±SD: 0.204 ± 0.288 and 0.213 ± 0.389), consistent with subclass levels measured during the clinical trial (14). IgG4 responses were sparse after dose 3, with only 10 of 94 samples (11%) above LOD and a mean ΔOD of 0.012 ± 0.043 (range: 0.00–0.269). Conversely, after dose 4, levels increased substantially, with detectable levels in 68 of 77 samples (88%) and a mean ΔOD of 0.823 ± 0.713 (range: 0.00–2.35).

C1q_F_ responses exhibited a bimodal distribution, with 1 subset of samples showing strong signal and another showing little to no response (Figure 2D). Although mean ΔOD values increased from 0.471 ± 0.867 after dose 3 to 0.639 ± 1.09 after dose 4, median values remained near LOD at both time points (Figure 2D). Only 49 of 87 samples (56%) after dose 3 and 36 of 60 (60%) after dose 4 had OD_Sera_ levels above the LOD. We examined calculating ΔOD as a percentage of total OD (%OD). ΔOD measurements (for IgG1, IgG3, IgG4, and C1q) strongly correlated with %OD (ρ = 0.58–1, all assays *P* < 0.0001, Supplemental Figure 5). In addition to ΔOD, %OD was explored in univariate analyses for predicting TRA (see below).

### P230Compete results correlate to TRA.

Next, the correlations of P230Compete levels to TRA (Supplemental Figures 2 and 6) were examined using Spearman’s rank-order testing (14). EU_F_ demonstrated the strongest association with TRA after dose 3 (ρ = 0.60) and after dose 4 (ρ = 0.57; both *P* < 0.0001). IgG1_F_ strongly correlated with TRA after dose 3 (ρ = 0.43, *P* < 0.0001) and after dose 4 (ρ = 0.42, *P* < 0.0001). Neither IgG3_F_ nor IgG4_F_ correlated with TRA after dose 3 or 4. C1q_F_ correlated strongly with TRA after dose 3 (ρ = 0.60, *P* < 0.0001), with moderate correlation after dose 4 (ρ = 0.39, *P* = 0.002). EU_TOTAL_ measured during the trial also correlated strongly with TRA after dose 3 (ρ = 0.46) and after dose 4 (ρ = 0.54, both *P* < 0.0001).

We examined the association between C1q_F_ responses and TRA (Supplemental Figure 7) by classifying samples as C1q-positive (defined as OD_Sera_ ≥ 0.5 and ΔOD ≥ 0.2) or C1q-negative. Given the known role of complement in Pfs230D1 vaccine activity (14–16), we compared the proportion of high responders (TRA ≥ 80%) between C1q strata using a χ^2^ test. After dose 3, 97% of C1q-positive samples exhibited TRA of 80% or higher versus 66% of the C1q-negative sample (*P* < 0.05), and 90% versus 59%, respectively, after dose 4 (*P* < 0.05), supporting an association between C1q_F_ positivity and high TRA.

Finally, we examined whether baseline TRA (measured by SMFA at day 0 prior to immunization) influenced P230Compete results (Supplemental Table 2). P230Compete results were stratified by participants with baseline TRA greater/equal to (*N* = 25 after dose 3 and *N* = 23 after dose 4) versus less than 80% (*n* = 69 after dose 3 and *n* = 54 after dose 4). No significant differences in mean P230Compete results were observed between participants with baseline TRA of 80% or higher and those without (<80%) at either post-dose 3 or post-dose 4 time points (all *P* > 0.05).

### P230Compete results predict TRA.

Building on the observed correlations with TRA, we next evaluated the ability of P230Compete results to predict high functional activity, defined as TRA of 80% or higher (Table 2). To do this, we performed univariate logistic regressions using ΔOD and ΔEU values, applying a training-testing model as described in Supplemental Figure 8, to assess the predictive performance of P230Compete for TRA levels from SMFA.

Excellent prediction was defined as AUC of 0.80 or higher and F1 scores of 0.70 or higher (33–35); F1 score (known as the harmonic mean of precision and recall) was reported, as SMFA datasets were skewed toward TRA of 80% or higher (Supplemental Figure 2). The P230Compete result with the lowest Akaike information criterion (AIC) value was considered the best model fit to TRA of 80% or higher. EU_F_ and IgG1_F_ reported AUC 0.80 or higher after doses 3 and 4 (Table 2). EU_F_ yielded an AUC of 0.81 after dose 3 and 0.81 after dose 4. IgG1_F_ demonstrated an AUC of 0.81 after dose 3 and 0.80 after dose 4, with F1 scores of 0.70 or higher at both time points. IgG3_F_ and IgG4_F_ were not predictive of TRA, with AUC less than 0.65 across time points (IgG3_F_ AUC of 0.60 after dose 3 and 0.62 after dose 4; IgG4_F_ AUC of 0.49 after dose 3 and 0.58 after dose 4) and F1 scores less than 0.7. C1q_F_ demonstrated AUC of 0.7 or higher, with post-dose 3 AUC of 0.82 and post-dose 4 AUC of 0.74, with F1 scores of 0.6 or higher across time points. Univariate prediction analysis was conducted for %OD results (Supplemental Table 3), which showed similar performance to ΔOD results.

EU_F_ and EU_TOTAL_ performed similarly based on AUC for both time points, with slightly higher AUC for EU_F_ after dose 3 and for EU_TOTAL_ after dose 4. A 2-sample, 2-tailed *t* test was applied to compare the difference in AUC between the 2 assays. After dose 3, the *P* value for the comparison between the 2 groups was 0.01, indicating a statistically significant difference between the AUCs of the 2 assay predictions. After dose 4, the *P* value for the comparison between the 2 groups was 0.09, which indicates a nonsignificant difference between the AUCs.

IgG1_F_ reported the lowest AICs after doses 3 and 4, indicating a stronger model fit to TRA compared with EU_F_, IgG3_F_, and IgG4_F_. For example, the AIC for IgG1_F_ was 10 lower in comparison to EU_F_ after dose 3, and values for both assays were similar after dose 4 (Table 2), indicating the better model fit to TRA of 80% or higher. IgG3_F_ and IgG4_F_ AIC values in comparison to EU_F_ and IgG1_F_ were 20–40 higher at both time points, indicating inferior model fit to TRA. Although C1q_F_ exhibited low AIC, this was not directly comparable to other P230Compete assays due to smaller sample size. EU_F_ achieved a lower AIC in comparison to EU_TOTAL_ after dose 3 (110 versus 123), and AIC values for both assays after dose 4 were similar. Our overall performance evaluation was based on multiple metrics (AIC, accuracy, F1, positive predictive value [PPV], negative predictive value [NPV], AUC, sensitivity, and specificity), rather than AUC alone. Considering all these measures, we conclude that P230Compete measurements demonstrate better predictive performance than EU_TOTAL_ for TRA of 80% or higher, especially after dose 3. In summary, EU_F_ and IgG1_F_ were the best univariate predictors of TRA.

### Optimal cutoffs for assays with excellent prediction of TRA of 80% or higher.

**Figure 3:**
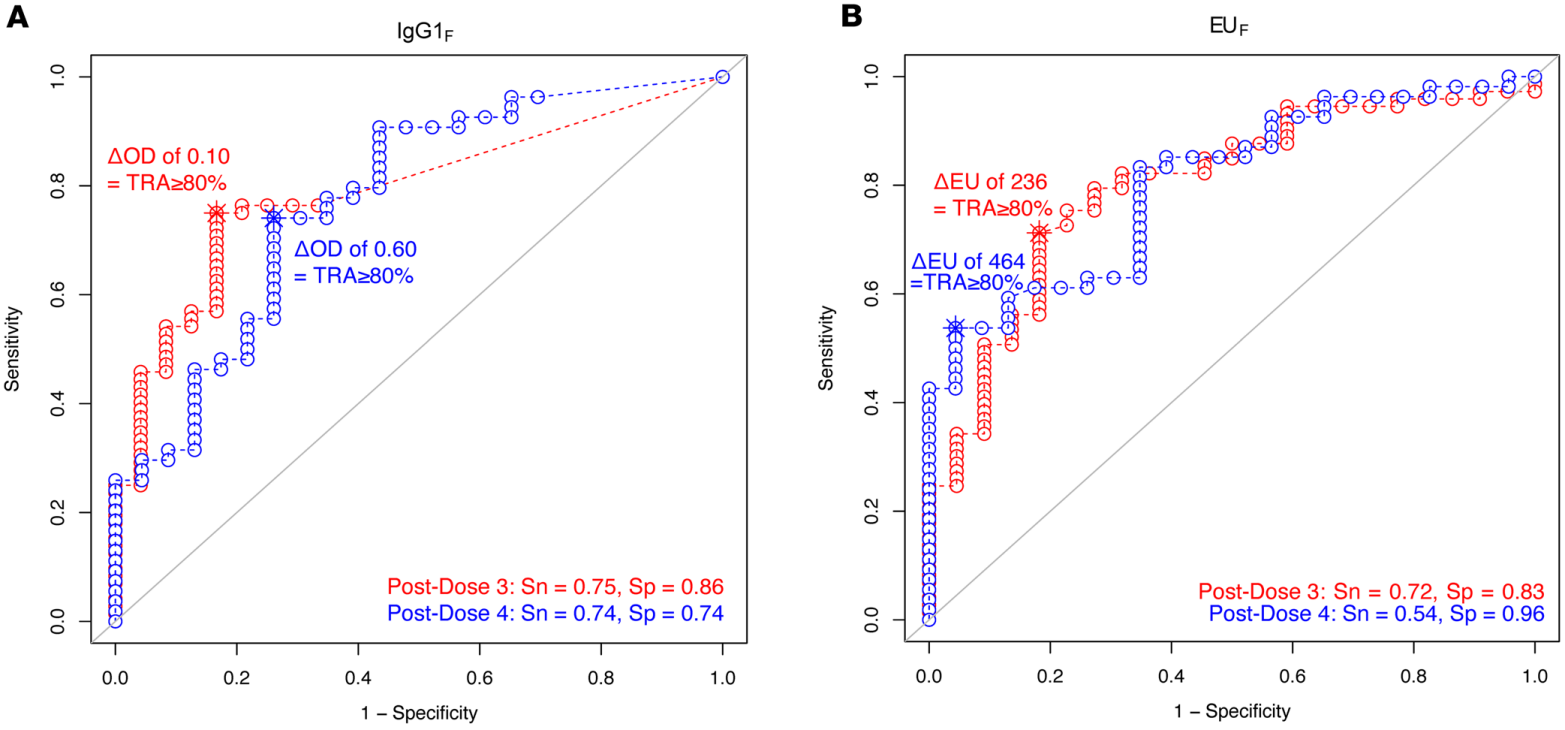
P230Compete prediction of TRA with optimal assay thresholds for TRA of 80% or higher. ROC curves for (**A**) IgG1_F_ (expressed as ΔOD) and (**B**) EU_F_ (expressed as ΔEU) analyzed after dose 3 (red) and after dose 4 (blue). Optimal assay cutoffs predictive of a TRA of 80% or higher calculated by Youden index. EU, ELISA units; Sn, sensitivity; Sp, specificity.

Next, to identify optimal assay cutoffs that were predictive of TRA of 80% or higher, we conducted ROC analysis. Optimal assay cutoffs were calculated by the Youden index and only evaluated for P230Compete assays with excellent prediction of TRA of 80% or higher, including IgG1_F_ and EU_F_ (Figure 3). The optimal cutoff for IgG1_F_ was ΔOD of 0.10 after dose 3 (sensitivity 0.75; specificity 0.86), and ΔOD of 0.60 after dose 4 (sensitivity 0.74; specificity 0.74) (Figure 3A). EU_F_ optimal assay cutoff was ΔEU of 236 after dose 3 (sensitivity 0.72; specificity 0.83), and ΔEU of 464 after dose 4 (sensitivity 0.54; specificity 0.96) (Figure 3B). In comparison, optimal assay cutoffs for EU_TOTAL_ were 252 EU after dose 3 (sensitivity 0.78; specificity 0.64) and 549 EU after dose 4 (sensitivity 0.54; specificity 1.0) (Supplemental Figure 9). ROCs were also analyzed for nonpredictive assays, including IgG3_F_, IgG4_F_, and C1q_F_, but since the AUCs were all below 0.80, optimal assay cutoffs were not calculated (Supplemental Figure 9).

### Combined assays better predict TRA of 80% or higher in comparison to a single assay.

To further investigate the relationship between P230Compete results and TRA, we next evaluated combinations of two P230Compete assays to predict TRA of 80% or higher. Pairwise logistic regressions were conducted on ΔOD and ΔEU values (Supplemental Table 4); the analysis did not include C1q_F_ results. The combination of IgG1_F_ and EU_F_ increased the prediction of TRA of 80% or higher in comparison to either single-marker performance: post-dose 3 AUC was 0.86 and post-dose 4 AUC was 0.87. IgG1_F_ and EU_F_ also achieved a lower AIC in comparison to either IgG1_F_ or EU_F_ alone. The combination of IgG1_F_ and EU_TOTAL_ increased the TRA 80% or higher prediction for post-dose 3 AUC to 0.85 and post-dose 4 AUC to 0.88. IgG1_F_ plus EU_TOTAL_ achieved a lower AIC in comparison to EU_TOTAL_ alone with 25 lower after dose 3 and 11 lower at after dose 4. Across these assay combinations, F1 scores were similar in comparison to single assay performance at both time points. Building on the improved performance observed with 2-assay combinations, we next evaluated whether incorporating a third or more P230Compete assays could further strengthen prediction of TRA of 80% or higher (Supplemental Table 5). The multivariate logistic regression model included EU_F_, IgG1_F_, IgG3_F_, IgG4_F_, EU_TOTAL_, sex, and age and excluded C1q_F_ results. Before running the multivariate model, multicollinearity of the P230Compete results was assessed as described in Supplemental Figure 10. Notably, EU_F_, EU_TOTAL_, IgG1_F_, and IgG3_F_ demonstrated statistically significant Spearman’s rank-order correlations ranging from 0.47 to 0.75 (all *P* < 0.05), with the IgG4_F_ dataset negligibly correlated with other assays. For the optimal assay models, the combination of EU_F_, IgG1_F_, and IgG3_F_ provided the best prediction performance after dose 3 with AUC of 0.85, F1 score of 0.81, and AIC of 92.8. After dose 4, the optimal model shifted to include IgG1_F_, IgG3_F_, and EU_TOTAL_ with AUC of 0.87, F1 score of 0.81, and AIC of 68.0. These 2 models outperformed most 2-assay combinations after dose 3 and after dose 4 except for IgG1_F_ plus EU_F_. Sex and age were included in the multivariate modeling but were not found to have a significant effect on prediction of TRA of 80% or higher and were not selected in either optimal multivariate model combination.

## Discussion

Here, we report on P230Compete, an ESC-ELISA platform designed to quantify antibody responses to functional epitopes on leading TBV candidate Pfs230D1. P230Compete uses scFvs that bind multiple functional epitopes on Pfs230D1 to quantify displacement of serum total IgG, IgG subclasses, and complement binding antibody against these epitopes. EU_F_ and IgG1_F_ measured by P230Compete achieved high correlation and excellent prediction of serum functional activity (defined as TRA ≥ 80%) measured in SMFA (AUC ≥ 0.8 and F1 ≥ 0.70), and improved prediction performance when combined. These findings suggest P230Compete may be useful as a surrogate assay for SMFA, which would enable high-throughput assessment of functional immunity in the field during large-scale trials.

As measurements of isotype subclasses and complement activation have been shown to be more predictive of functional antibody responses in comparison to total IgG titers (15, 16, 27), P230Compete was designed to measure both complement-fixing (IgG1, IgG3) (12, 14) and non-fixing IgG (IgG4) subclasses, in addition to direct C1q binding. When applied to sera collected during a phase I trial of Pfs230D1, P230Compete results were consistent with prior preclinical and clinical trial results, which have shown Pfs230D1 vaccine activity to be complement dependent and IgG1 to be a dominant antibody subclass (12–14). IgG1_F_ consistently outperformed IgG3_F_ and IgG4_F_ in both correlation to and prediction of TRA. Although IgG3_F_ did not independently predict TRA, its inclusion in the multivariate models suggests potential value as part of a composite assay panel to predict TRA. In contrast, IgG4_F_ failed to predict TRA, despite high responses after dose 4, suggesting it may have a limited role in Pfs230D1 serum activity.

Levels of C1q_F_, a marker of antibody-mediated complement activation, also correlated with TRA. As the first effector molecule of the classical complement cascade, C1q plays a critical role in initiating membrane attack complex formation and parasite lysis (16). Samples with detectable levels of C1q_F_ (ΔOD ≥ 0.2, OD_Sera_ ≥ 0.5) were more likely to exhibit TRA of 80% or higher, consistent with the role of complement in functional activity of Pfs230D1 anti-sera. Although C1q_F_ did not out-perform EU_F_ and IgG1_F_ in univariate prediction of SMFA activity, the results presented here show promise for use of C1q_F_ as a correlate of high TRA. Further assay optimization and a larger sample size may improve its performance in future studies.

P230Compete builds on the expanding field of ESC-ELISAs, which have been developed for other malaria antigens (31, 32, 36, 37), such as apical membrane antigen 1 (AMA1), circumsporozoite protein, and Pfs48/45. These earlier platforms measured competition at a single functional epitope using biotinylated hmAbs (e.g., AMA1 with 4G2) or plate-bound competitors (e.g., TB31F for Pfs48/45) (31, 32). In contrast, P230Compete uses scFvs that target multiple nonoverlapping functional epitopes to block an entire surface of Pfs230D1, allowing a more comprehensive assessment of serum functional activity. This approach was guided by recent structural mapping of epitopes on Pfs230D1 (15), which identified the generally conserved region of the functional face. This antigen surface is the shared target of multiple transmission-blocking hmAbs, underscoring this region of Pfs230D1 as a critical driver of serum functional activity.

By interrogating multiple functional epitopes simultaneously, P230Compete overcomes key limitations of single-epitope ESC-ELISAs. For example, in the Pfs48/45 ESC-ELISA, total IgG levels were a better predictor of TRA than the single epitope-specific ESC-ELISA measurement for a subset of samples (32). This result might be explained if additional functional epitopes not detected by the assay contribute to TRA. P230Compete design addresses this by capturing polyclonal functional antibody responses and potential synergistic Pfs230D1 antibody effects (15). Furthermore, the use of scFvs, which lack Fc regions, allows for measurement in sera of the isotype, subclass, or complement fixing features that target the blocked epitope.

While promising, P230Compete has limitations. It cannot fully replicate the biological complexity of mosquito-parasite interactions captured by SMFA (26, 38). The assay requires larger serum volumes than standard ELISA, as samples must be tested with and without scFv to calculate ΔOD and ΔEU values. With 10-fold less antigen absorbed on P230Compete plates, in comparison to direct ELISA, sensitivity is reduced for low-abundance isotypes like IgG3 and IgG4. Furthermore, the current findings, although promising, are based on a single Pfs230D1 trial in adults and limited to 2 time points, underscoring the need for studies at different sites to improve generalizability to malaria-endemic populations.

Future studies analyzing sera from multiple Pfs230D1 trials could further refine which P230Compete assays best predict TRA and in what combinations. Additionally, the scFv-based ESC-ELISA platform targeting multiple functional epitopes could be adapted to other TBV antigens to assess polyclonal functional antibody responses. In summary, P230Compete is a scalable, high-throughput assay that combines IgG-, subclass-, and complement-specific ESC-ELISAs to predict TRA measured by SMFA. As Pfs230D1-EPA/AS01 progresses in development, P230Compete can be a valuable tool to assess serum functional activity in the high number of samples required for late-stage trials and thereby accelerate TBV development, conserve resources, and serve as a proxy endpoint for SMFA. By enabling real-time assessment of functional immunogenicity, P230Compete may provide a practical and scalable surrogate endpoint in lieu of SMFA to guide vaccine evaluation and accelerate TBV development.

## Methods

### Sex as a biological variable.

Serum samples analyzed from the Pfs230D1 trial were predominantly from male participants (70% after dose 3 and 71% after dose 4), due to various trial-specific factors of eligibility criteria (e.g., exclusion of pregnant women and contraception requirements for safety reasons) (14). Biological sex was included as a covariate in the multivariate model to mitigate confounding but did not markedly affect immunological outcomes and was not selected for the final models.

### Human participant research approval.

The trial protocol (ClinicalTrials.govNCT02942277) was approved by ethics committees (U.S. National Institute of Allergy and Infectious Diseases; Mali Faculté de Médecine, de Pharmacie et d’OdontoStomatologie) and the Mali national regulatory authority. The study was conducted under FDA IND 17130, in accordance with International Council for Harmonization Good Clinical Practice guidelines and applicable local regulations. Written informed consent was obtained in the local languages, with approval from community leaders as reported previously (14).

### Pfs230D1 trial design.

Samples analyzed by the P230Compete panel assay were from a phase I randomized, double-blind, comparator-controlled clinical trial (ClinicalTrials.gov NCT02942277) conducted between 2017 and 2018 in Bamako and Bancoumana, Mali, as reported previously (14). The study evaluated the safety and immunogenicity of Pfs230D1-EPA/AS01 in healthy adults aged 18–50 years, with exclusion criteria for pregnant or breastfeeding women, and 245 adults were randomized into 3 arms: full group (*n* = 56), fractional group (*n* = 61), and comparator arm (*n* = 119). The trial consisted of 7 study days with vaccinations administered at months 0, 1, 4, and 16. Full-group participants received 40 μg per dose at all time points. At the third vaccination (month 4), the fractional group received a reduced 8 μg dose. Comparator participants received ENGERIX-B (hepatitis B vaccine) at months 0, 1, and 4, and Menactra (a meningococcal vaccine covering serogroups A, C, Y, and W-135) for month 16 as a fourth booster.

### Plasmid design and cloning of hmAb and scFv constructs.

Plasmids encoding hmAbs and scFv constructs for LMIV230-01, AS-18, and AL-18 were generated as previously described (15). Heavy and light chain variable regions (synthesized by GenScript) were cloned into a human IgG1 expression plasmid (InvivoGen, pfusess-hchg1e1, pfuse2ss-hclk, pfuse2ss-hcll2). Heavy chains were cloned between a signal peptide and the constant region; light chains were cloned as either kappa or lambda isotypes based on their native sequence. For scFv constructs, the heavy and light chain variable regions were linked using a (GGGGS)_4_ flexible linker and cloned downstream of a signal peptide into the pHLSEC expression plasmid.

### Cell culture and transfection.

Expi293 suspension cells (Thermo Fisher Scientific, A14527) were cultured in Expi293 expression medium (A1435101) at 37°C with 8% CO_2_ and maintained in log-phase growth (3.0 × 10^6^ to 5 × 10^6^ cells/mL) for 3–4 passages after thawing. For transfection, cells were seeded the day before at 2.5 × 10^6^ to 3×10^6^ cells/mL in 500 mL volumes using 2 L flasks. On the day of transfection, cell density was rechecked and adjusted as needed. Transfection was carried out using 0.5 mg of plasmid DNA and 1.4 mL of ExpiFectamine (Thermo Fisher Scientific, A14525) per 500 mL culture. DNA and ExpiFectamine were each diluted in 25 mL Opti-MEM (catalog 31985062), with DNA passed through a 0.2 μm filter. After a 5-minute incubation, the 2 solutions were combined, incubated for 10–20 minutes at room temperature, and added slowly to the culture while swirling. Cultures were returned to 37°C/8% CO_2_ for 16–20 hours. The next day, Enhancer I and II (Thermo Fisher Scientific, A39249) were added, and cultures were maintained under the same conditions. After 5 days, cells were harvested by centrifugation at 5,000*g* for 30 minutes, and supernatants were clarified via sequential 0.2 μm filtration.

### hmAb expression and purification.

Clarified media was passed through a 5 mL Protein A Trap column (Cytiva Life Sciences, 17040303), washed with 20-column volumes of binding buffer (Thermo Fisher Scientific, 2100100), and eluted with IgG buffer (Thermo Fisher Scientific, 21004). The eluted antibody was immediately neutralized with 1/5 volume of 1 M Tris-HCl (pH 9), buffer-exchanged into PBS (pH 7.4) via dialysis, and the concentration was quantified by absorbance at 280 nm using an extinction coefficient of 1.4. Purified LMIV230-01, AS-18, and AL-18 antibodies (produced at NIH/LMIV by previously described methods ([15, 16]) were aliquoted and stored at –80°C.

### scFv expression and purification.

Clarified supernatants were loaded on a 5 mL HisTrap Excel NTA column (Cytiva, 17371206), washed with 20-column volumes of buffer (20 mM sodium phosphate, 0.5 M NaCl, 30 mM imidazole, pH 7.4), and eluted using a step gradient of 0.5 M imidazole in elution buffer containing 0.5 M L-arginine (pH 7.4). Eluted fractions were analyzed by SDS-PAGE (4%–12% Bis-Tris; Thermo Fisher Scientific, NP0322BOX) and Coomassie staining. scFv-positive fractions were pooled, concentrated using 10 kDa cutoff centrifugal filters (MilliporeSigma, MRCPRT010), and further purified by SEC on a Sepharose S200 column (Cytiva, 28990946) equilibrated in PBS plus 0.1 M L-arginine (pH 7.4). Final scFv fractions were pooled, concentrated, and quantified by A280 using sequence-based extinction coefficients, aliquoted, and stored at –80°C. This protocol was used to generate scFv_LMIV230-01_, scFv_230AL-18_, and scFv_230AS-18_.

### Pfs230 expression and purification.

Recombinant Pfs230 was expressed in *Pichia pastoris* using a codon-optimized gene construct. Expression and purification were performed following previously described methods (12, 13, 39).

### C1q biotinylation and tetramer assembly.

Monomeric C1q (Complement Technology, A099) was biotinylated with Sulfo-NHS-LC-LC-biotin (Thermo Fisher Scientific, A35358) at a 20:1 molar ratio of biotin/C1q for 3 hours at 4°C. The protein was buffer-exchanged into 10 mM HEPES (pH 7.2), 300 mM NaCl, and 40% glycerol. For tetramer assembly, biotinylated C1q was added incrementally (1/10th volume every 10 minutes) to streptavidin-HRP (Jackson ImmunoResearch, 016-030-084) at a 4:1 molar ratio and stored at 4°C until use.

### Competition ELISA (P230Compete) using scFvs against Pfs230D1 functional face.

Immulon 4 HBX plates (Thermo Fisher Scientific, 3855) were coated overnight at 4°C with 0.1 μg/mL Pfs230D1M in carbonate buffer (pH 9.6). The coating concentration for P230Compete was a 10-fold reduction compared with 1.1 μg/mL coating used in direct ELISAs to measure Pfs230D1M titers during the trial, as reported previously (14). The reduction in antigen coating for P230Compete minimized scFv reagents required to saturate the functional face of Pfs230D1. Plates were blocked with PBST plus 5% powdered dry milk (PDM) for 30 minutes at room temperature and washed with PBST (G Biosciences, R044). For the total IgG assay, sera were diluted 1:500 in PDM and incubated with or without 10 μg/mL each of scFv (scFv_LMIV230-01_, scFv_230AL-18_, scFv_230AS-18_) for 1 hour. Plates were washed (4 times) and bound total IgG (EU_F_) was detected using HRP-conjugated anti-human IgG (1:20,000, Jackson ImmunoResearch, 109-035-003), followed by 10-minute 3,3′,5,5′-Tetramethylbenzidine (TMB) development in the dark. Absorbance was measured at 450 nm (SpectraMax 340 PC). EUs were calculated using a 4-parameter logistic fit to plate-specific standard curves generated from a serial dilution of pooled high-titer sera of 11 trial participants from month 17. For IgG isotyping (IgG1_F_, IgG3_F_, IgG4_F_) assays, the protocol followed that of total IgG with the following modifications: sera were diluted 1:250 (IgG1 and IgG4) and 1:50 (IgG3) and incubated with or without 10 μg/mL of each scFv (scFv_LMIV230-01_, scFv_230AL-18_, scFv_230AS-18_) for 2 hours. TMB development was extended to 16 minutes. Detection was performed using anti-human subclass-specific secondary antibodies: IgG1 (1:300, Invitrogen, A10648), IgG3 (1:1,200, Sigma Aldrich, SAB4200769-1VL), and IgG4 (1:1,200, Thermo Fisher Scientific, A10654). The standard for the P230Compete IgG1 assay was recombinant LMIV230-01. For the C1q (C1q_F_) assay, plates were coated with 1.1 μg/mL Pfs230D1-EPA. Sera were diluted 1:50 and incubated with or without each scFv (scFv_LMIV230-01_, scFv_230AL-18_, scFv_230AS-18_) for 1 hour. Next, 4 μg/mL biotinylated C1q tetramer (secondary) was incubated for 1 hour. The plate standard matched that of the EU_F_ assay with a pooled high-titer sera (11 participants).

### Pfs230D1 samples analyzed by panel of P230Compete assays.

Sera samples analyzed by the P230Compete assay panel included 75 paired samples (defined as participants with samples at both time points that included 38 participants from the full-dosing group and 37 participants from the fractional-dosing group). In addition to the paired samples, there were 19 participants with only post-dose 3 samples (10 participants from the full-dosing group and 9 from the fractional-dosing group), and 2 participants with only post-dose 4 samples (from the fractional-dosing group). Full and fractional groups were combined for TRA prediction analyses as baseline demographics were matched between cohorts and TRA values were similar as reported previously (14). The C1q assay was limited by insufficient sample volume remaining for analysis, with 87 of 94 (93%) post-dose 3 samples (5 participants from the full-dosing group and 2 from the fractional-dosing group) and 60 of 77 (78%) post-dose 4 samples (8 participants from full-dosing group and 9 participants from the fractional dosing-group). The IgG3 assay had 1 sample with insufficient volume after dose 3 (from the full-dosing group) with a total of 93 of 94 (99%) samples analyzed and all available samples (77 of 77 samples) analyzed after dose 4. Six comparator participants were randomly assigned across assay plates to serve as negative controls and establish background reactivity. Sera from these same individuals were analyzed after dose 3 and after dose 4 for a total of 12 comparator arm samples analyzed across the P230Compete assay panel.

### P230Compete measurements: OD_Sera_ levels for pilot study of sera samples analyzed.

After optimizing the P230Compete platform, the ESC-ELISA was expanded to 5 assay format measuring antibody markers, including complement-fixing and non-complement fixing IgG subclasses and direct C1q complement binding. Measurements included EU_F_ (total IgG); IgG isotypes (IgG1_F_, IgG3_F_, IgG4_F_); and C1q_F_, in which “F” was defined as antibody displacement to the functional face expressed as ΔOD or for total IgG (ΔEU) (Figure 1D). To identify optimal assay conditions for each individual P230Compete assay, serum samples were diluted across a range from 1:50 to 1:3,000 to identify OD levels in the linear ranges for each assay.

Based on these titrations, final working dilutions were selected: 1:500 for total IgG, 1:250 for IgG1 and IgG4, and 1:50 for IgG3 and C1q; attempts to develop a pooled standard of IgG2 and measure epitope-specific displacement of IgG2 were unsuccessful owing to low levels of IgG2, also observed by standard isotyping ELISA of trial samples (14). Dilutions were lower for IgG isotyping and C1q due to lower isotype-specific antibody titers measured during the Pfs230D1 trial, as reported previously (14). Sera samples after doses 3 and 4 were analyzed across the P230Compete assay panel with 5 outcome values: EU_F_ (total IgG), IgG1_F_, IgG3_F_, IgG4_F_, and C1q_F_. Each sera sample was analyzed in duplicate with 2 outcome values (OD_Sera_ and OD_Sera+scFv_). Arithmetic means and coefficients of variation were calculated, and coefficients of variation of 20% or less were considered acceptable. Sera samples with OD_Sera_ values above the LOD, ΔOD, and %OD were calculated. LOD was defined by a pool of O+ malaria–naive sera of 22 heat-inactivated samples. The plate-specific LOD was defined as the mean of 4 replicates plus 3 SD. Samples with OD_Sera_ values below the LOD at the working dilutions were re-assayed at a lower dilution (more concentrated) to improve detection for low titer samples. Samples with OD_Sera_ values below the LOD once re-assayed were considered nonreactive to Pfs230D1, and ΔOD and %OD values were set to zero.

In the P230Compete Total IgG assay, all serum samples elicited detectable OD_Sera_ signal at the 1:500 working dilution, with no re-assaying needed. Similarly, EU_Sera_ levels for all samples were above the LOD; thus, ΔEU values were calculated for every sample. In contrast, IgG isotypes exhibited greater variability in OD_Sera_ levels, particularly IgG1 and IgG4, requiring re-assaying of several samples (Table 1). After dose 3, 59 of 94 IgG1 samples (63%) required re-assay at 1:50, with 31 (33%) still undetectable. After dose 4, 30 of 77 samples (39%) were re-assayed, and 9 (12%) remained undetectable. IgG3 assays were run with sera 1:50, with 29 of 94 (31%) and 21 of 77 (27%) undetectable at months 9 and 20, respectively. IgG4 showed the lowest reactivity: 84 of 94 samples (89%) after dose 3 required re-assay at 1:50, with all 84 remaining undetectable; after dose 4, 33 of 77 (43%) were re-assayed, and 9 (12%) remained undetectable. C1q assay analysis was limited by insufficient volume remaining for analysis with 87 of 94 (93%) samples after dose 3 and 60 of 77 (78%) samples after dose 4 available. Of these, 36 (42%) and 24 (40%) had undetectable OD_Sera_ values at 1:50 working dilution. For all undetectable samples (below the malaria-naive LOD), ΔOD and %OD values were set to zero. For comparators, all OD_Sera_ values were below the LOD across the assay panel; thus, comparator analysis was not included in TRA analyses.

### P230Compete ΔOD and %OD calculations and data processing.

For the P230Compete assays, serum antibody displacement from the functional face of Pfs230D1 was quantified using 2 OD levels: OD_Sera_ (with serum only) and OD_Sera+scFv_ (with serum plus saturating scFv) (Figure 1C). Serum antibody displacement from the functional face of Pfs230D1 was calculated for 2 measurements (ΔOD and %OD):

For the total IgG assay (EU_F_), OD values were converted to EUs using a 4-parameter logistic curve generated from a pool of high-titer sera from the trial (month 17). After converting OD values to EU, ΔEU and %EU values were similarly calculated as ΔOD:

Because low denominator values may skew %OD and %EU values, ΔOD and ΔEU were selected a priori as the primary P230Compete outcome for downstream analyses and predictive modeling and compared with %OD for performance.

### SMFA conducted during the Pfs230D1 trial.

SMFA was conducted on sera collected from participants enrolled in the Pfs230D1 malaria vaccine trial. In each assay, test sera obtained from immunized individuals was used neat and mixed with cultured *P*. *falciparum* (NF54 strain) gametocytes, prepared from 14–16-day in vitro cultures containing mature stage 5 gametocytes. The serum-parasite mixture was transferred into a glass feeder cup covered with an artificial membrane and maintained at 40°C by circulating warm water. Laboratory-reared *Anopheles stephensi* mosquitoes (Nijmegen strain), previously starved for approximately 24 hours, were allowed to feed through the membrane for 20 minutes. A parallel control feed using malaria-naive human serum was conducted under identical conditions using mosquitoes from the same colony. After the blood meal, mosquitoes were maintained at 27°C and 80% humidity for 8 days to allow for parasite development. Midguts from at least 20 mosquitoes per group were dissected, stained with 0.05% mercurochrome, and examined microscopically for oocyst enumeration. Feeding assays were considered valid only if the average oocyst counts in control mosquitoes exceeded 4. TRA was calculated as [1 − (mean oocyst count in test group / mean oocyst count in control group)] × 100.

### Statistical interpretation of P230Compete prediction of functional serum activity (TRA).

Three logistic regression models were developed to evaluate the ability of P230Compete assay results to predict TRA of 80% or higher: univariate (single assay: Table 2), pairwise (2 assays: Supplemental Table 4), and multivariate models (3 or more assays including sex and age: Supplemental Table 5). TRA was classified as a binary outcome of greater and less than 80% in logistic regression analyses. The following P230Compete results were included in the models as continuous variables: ΔEU for total IgG (EU_F_) and ΔOD for IgG isotypes and complement binding (IgG1_F_, IgG3_F_, IgG4_F_, and C1q_F_). The statistical workflow and model construction are detailed in Supplemental Figure 8. Univariate analysis was conducted for both ΔOD/ΔEU and %OD/%EU results (Table 2 and Supplemental Table 3). SMFA datasets used for TRA prediction were from phase I of the Pfs230D1 trial (ClinicalTrials.gov NCT02942277) as reported previously (14). The multivariate model included the following variables: EU_F_, IgG1_F_, IgG3_F_, IgG4_F_, C1q_F_, EU_TOTAL_, age, and sex (Supplemental Table 5). Performance statistics included AUC (40), AIC, PPV, NPV, and F1 score. Prediction results with AUC of 0.80 or higher and F1 scores of 0.70 or higher from the test set were considered excellent (33–35). Best model fit was evaluated by the lowest AIC, with a lower AIC indicating better model fit to predict TRA of 80% or higher. Optimal P230Compete assay cutoffs were determined by the Youden index for a TRA of 80% or higher for assays with AUC of 0.80 or higher (Figure 3); optimal assay cutoffs were not calculated for P230Compete assays with AUC less than 0.80 (Supplemental Figure 9).

### Statistics.

All analyses were performed in R (version 4.3.3). Summary statistics were calculated using the arsenal package (41), applying χ^2^ tests for categorical variables and 2-sample, 2 tailed *t* tests for continuous variables. The Shapiro-Wilk test was used to assess the normality of P230Compete and EU_TOTAL_ datasets. For ELISA result comparisons, log-transformed EU and OD values were used for all analyses. Binary values were assigned to TRA, and total IgG datasets (EU_F_ and EU_TOTAL_) were log-transformed for univariate, pairwise, and multivariate analysis of ΔOD. For OD_Sera_ levels below the LOD, ΔOD were not calculated and were set to zero. For %OD univariate analysis, total IgG datasets (EU_F_ and EU_TOTAL_) were square-root transformed. Spearman’s correlations were evaluated between P230Compete OD_Sera_ values and Pfs230D1 titers (14) (Supplemental Figure 4). Correlations were also assessed between ΔOD and %OD, as well as ΔEU and %EU (Supplemental Figure 5). Lastly, correlations between P230Compete results and TRA were analyzed (Supplemental Figure 6). A linear regression model was fitted using the least-squares method. We calculated 95% CI as the predicted value ± *t* × SE, where *t* is the critical value and SE is the standard error of the predicted mean response. Univariate and multivariate logistic regressions for TRA prediction were constructed as detailed in Supplemental Figure 8 and as detailed in the Methods. C1q_F_ results were excluded from multivariate logistic regressions due to limited sample size. Baseline demographics of age and sex were included in multivariate logistic regressions in identifying optimal assay combinations of 3 or more markers. ROCs were generated with “pROC” R package (version 1.18.5) to assess classification performance for TRA of 80% or higher. Optimal assay threshold for TRA of 80% or higher was calculated for assays with AUC of 0.80 or higher determined by the Youden index (Figure 3). Optimal assay cutoffs were not calculated for P230Compete assays with AUC less than 0.80 (Supplemental Figure 9).

### Data availability.

Data used in the compilation of figures presented in this article and the supporting code are supplied in the supplemental information. The supporting code is included in the public GitHub repository (https://github.com/niaid/cELISA-StatisticalAnalysis).

## Author contributions

CAM optimized, performed, and analyzed P230Compete assays and wrote the first manuscript draft. PED and JPR conceived the assay platform, mentored the project, and revised the manuscript draft. JPR and MVC generated recombinant reagents and optimized a competitive ELISA. RDM processed and analyzed datasets. YY, JG, and MPP performed statistical analysis. SAH and IS supervised the Pfs230D1-EPA/AS01 clinical trial. JCCH supervised SMFA analyses. All authors contributed to manuscript generation and review.

## Funding support

This research was supported by the Intramural Research Program of the NIH. This work is the result of NIH funding, in whole or in part, and is subject to the NIH Public Access Policy. Through acceptance of this federal funding, the NIH has been given a right to make the work publicly available in PubMed Central.

- Division of Intramural Research (DIR) of the National Institute of Allergy and Infectious Diseases (NIAID), NIH.
- DHHS and with federal funds from the NIAID, NIH.
- DHHS under Bioinformatics and Computational Biosciences Branch, NIAID.
- Support Services Contract HHSN316201300006W/75N93022F00001 to Guidehouse Digital.
- DIR/NIAID/NIH (to CAM) for participation in Office of Intramural Training and Education Graduation Partnership Program.
- Medical Scientist Training Program at Heersink School of Medicine, University of Alabama at Birmingham.

## Supplementary Material

Supplemental data

Supplementary Figures and Tables

Supporting data values

## Figures and Tables

**Table 1:**
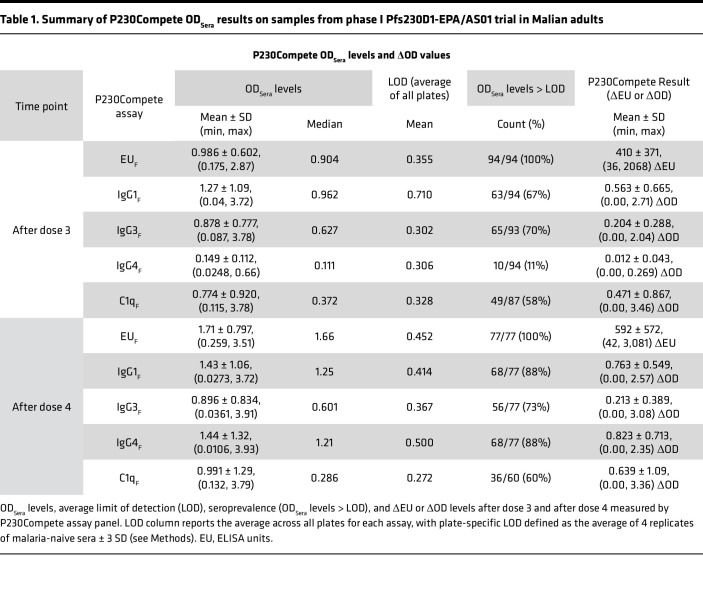
Summary of P230Compete OD_Sera_ results on samples from phase I Pfs230D1-EPA/AS01 trial in Malian adults

**Table 2:**
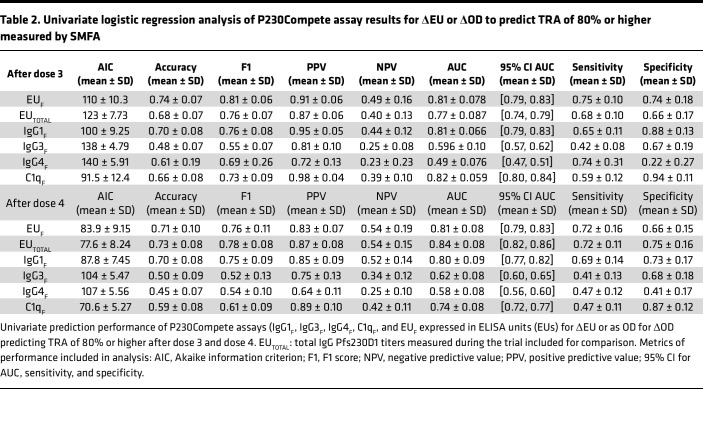
Univariate logistic regression analysis of P230Compete assay results for ΔEU or ΔOD to predict TRA of 80% or higher measured by SMFA

## Version 1 01/01/2026

In-Press Preview

## Version 2 02/09/2026

Electronic publication
