# Supplementary material for: Epitope-specific competitive ELISA predicts malaria transmission-blocking vaccine Pfs230D1 activity measured in standard membrane feeding assay
Source: JCI Insight. 2026 Jan 1;11(3):e198414. doi: 10.1172/jci.insight.198414 (PMC12892893; doi:10.1172/jci.insight.198414)
Supplement: Supplemental data [file jciinsight-11-198414-s169.pdf]

Supplementary Materials for

Epitope-specific competitive ELISA predicts malaria transmission-blocking vaccine Pfs230D1 activity measured in standard membrane feeding assay

Cristina A. Meehan[1,2], Matthew V. Cowles[1], Robert D. Morrison[1], Yuyan Yi[3], Jingwen Gu[3], Jen. C. C. Hume[1], Mina P. Peyton[3], Issaka Sagara[4,5], Sara A. Healy[1], Jonathan P. Renn[1], and Patrick E. Duffy[1]
Correspondence to: patrick.duffy@nih.gov

Supplementary Tables:

1. Summary of transmission reducing activity by timepoint, dosing group and TRA≥80% subgroups.
2. P230Compete assay panel results stratified by baseline TRA.
3. Univariate logistic regression of single P230Compete assay results for %EU or %OD to predict TRA≥80%.
4. Pairwise logistic regression of two P230Compete assay results for ΔEU or ΔOD to predict TRA≥80%.
5. Multivariate logistic regression of three or more P230Compete assay results for ΔEU or ΔOD to predict TRA≥80%.

Supplementary Figures:

1. Human serum does not impact the blocking function of scFv.
2. TRA datasets used in P230Compete prediction analyses.
3. Distribution of EU_{Sera} and OD_{Sera} results across P230Compete assay panel post-dose three and four.
4. Correlation between P230Compete OD_{Sera} results and Pfs230D1 titers measured by direct ELISA.
5. Correlation between the two P230Compete measurements of percent reduction and delta reduction.
6. P230Compete results correlated to TRA.
7. $C1q_F$ levels differentiate high versus low TRA post-vaccination.
8. Workflow for statistical prediction model construction and analysis.
9. P230Compete prediction of TRA by receiver operator characteristic curves.
10. Correlation analysis of P230Compete results to assess multicollinearity for multivariate modeling.

Table S1. Summary of transmission reducing activity by timepoint, dosing group and TRA≥80% subgroups.

Analysis	Timepoint	Group	N	TRA Mean ± SD, (Min, Max)	TRA Median	P-value
All Participants	Post-Dose 3	All	94	84.6 ± 23.4 (0.00, 100)	95.9	p=0.838
	Post-Dose 4	All	77	84.3 ± 22.7 (0.00, 100)	94.7	
	Timepoint	Group	Count (%)	TRA Mean ± SD, (Min, Max)	TRA Median	
TRA≥80% Subgroup	Post-Dose 3	All (subset)	25/94 (27%)	94.9 ± 5.71, (80.8, 100)	98.6	
	Post-Dose 4	All (subset)	22/77 (29%)	94.8 ± 5.82, (80.8, 100)	97.7	
	Timepoint	Group	N	TRA Mean ± SD, (Min, Max)	TRA Median	P-value
Pfs230D1 Dosing Groups	Post-Dose 3	Full	48	89.3 ± 19.4, (3.90, 100)	95.8	p=0.074
	Post-Dose 3	Fractional	46	79.8 ± 26.2, (0.00, 100)	94.0	
	Post-Dose 4	Full	38	83.2 ± 24.0, (0.00, 100)	94.7	p=0.984
	Post-Dose 4	Fractional	39	85.3 ± 21.5, (2.70, 100)	96.0	

Transmission reducing activity (TRA) datasets measured by standard membrane feeding assay (SMFA) during the Pfs230D1 trial as reported in [14]. Results presented for all participants in the P230Compete analysis by timepoint, subgroup of participants with TRA≥80%, and stratified by full and fractional dosing groups. Statistical comparisons of median rank of TRA values post-dose 3 and post-dose 4 were performed using the Mann–Whitney U test, as well as for data stratified by full versus fractional dosing groups.

Table S2. P230Compete assay panel results stratified by baseline TRA.

Post-Dose 3 Results			
	Group 1: Baseline TRA≥80%	Group 2: No Baseline TRA<80%	
	TRA≥80%, Count (%)	TRA<80%, Count (%)	
Total Samples N=94	25 (27%)	69 (73%)	
Standard Membrane Feeding Assay			
	Mean ± SD (Min, Max)	Mean ± SD (Min, Max)	p-value
TRA (%)	94.9 ± 5.71 (80.8, 100)	37.2 ± 27.4 (0, 78.1)	
Total IgG			
EU_F (ΔEU)	375 ± 335 (50, 1444)	418 ± 384 (36, 2068)	0.595
EU_{TOTAL} (EU)	380 ± 189 (89, 799)	447 ± 445 (84, 2730)	0.306
IgG subclass and complement binding			
$IgG1_F$ (ΔOD)	0.59 ± 0.59 (0.00, 2.04)	0.54 ± 0.69 (0.00, 2.71)	0.733
$IgG3_F$ (ΔOD)	0.16 ± 0.19 (0.00, 0.69)	0.22 ± 0.32 (0.00, 2.03)	0.33
$IgG4_F$ (ΔOD)	0.01 ± 0.06 (0.00, 0.27)	0.01 ± 0.04 (0.00, 0.25)	0.94
$C1q_F$ (ΔOD)	0.42 ± 0.78 (0.00, 2.74)	0.48 ± 0.90 (0.00, 3.46)	0.752
Post-Dose 4 Results			
	Group 1: Baseline TRA≥80%	Group 2: No Baseline TRA<80%	
	TRA≥80%, Count (%)	TRA<80%, Count (%)	
Total Samples N=77	23 (30%)	54 (70%)	
Standard Membrane Feeding Assay			
	Mean ± SD (Min, Max)	Mean ± SD (Min, Max)	
TRA (%)	94.8 ± 5.82 (80.8, 100)	38.4 ± 28.0 (0, 78.1)	
Total IgG			
EU_F (ΔEU)	531 ± 387 (42, 1746)	588 ± 637 (58, 3081)	0.631
EU_{TOTAL} (EU)	610 ± 494 (97, 2340)	616 ± 787 (61, 5132)	0.97
IgG subclass and complement binding			
$IgG1_F$ (ΔOD)	0.77 ± 0.45 (0.00, 1.70)	0.76 ± 0.59 (0.00, 2.57)	0.959
$IgG3_F$ (ΔOD)	0.17 ± 0.22 (0.00, 0.77)	0.23 ± 0.44 (0.00, 3.08)	0.457
$IgG4_F$ (ΔOD)	0.68 ± 0.69 (0.00, 2.00)	0.88 ± 0.72 (0.00, 2.35)	0.237
$C1q_F$ (ΔOD)	1.09 ± 1.38 (0.00, 3.28)	0.45 ± 0.89 (0.00, 3.36)	0.084

P230Compete results stratified into two groups based on TRA (TRA≥80% vs <80%). EU: ELISA units. P230Compete results for total IgG reported as ΔEU and IgG isotyping and complement binding reported as ΔOD. Continuous results analyzed by Welch's t-test.

Table S3: Univariate logistic regression of single P230Compete assay results for EU, %EU or %OD to predict TRA≥80%.

Post-Dose 3									
	AIC (Mean ± SD)	Accuracy (Mean ± SD)	F1 (Mean ± SD)	PPV (Mean ± SD)	NPV (Mean ± SD)	AUC (Mean ± SD)	95%CI (AUC)	Sensitivity (Mean ± SD)	Specificity (Mean ± SD)
EU_F	108 ± 10.6	0.70 ± 0.08	0.77 ± 0.072	0.91 ± 0.057	0.44 ± 0.15	0.81 ± 0.08	[0.79, 0.83]	0.67 ± 0.12	0.78 ± 0.17
EU_{TOTAL}	122 ± 7.61	0.65 ± 0.07	0.73 ± 0.064	0.87 ± 0.061	0.37 ± 0.12	0.77 ± 0.09	[0.74, 0.79]	0.64 ± 0.09	0.67 ± 0.17
$IgG1_F$	111 ± 8.57	0.72 ± 0.08	0.78 ± 0.071	0.93 ± 0.055	0.45 ± 0.12	0.79 ± 0.08	[0.77, 0.81]	0.68 ± 0.10	0.82 ± 0.15
$IgG3_F$	141 ± 5.04	0.49 ± 0.05	0.60 ± 0.065	0.75 ± 0.083	0.21 ± 0.10	0.58 ± 0.08	[0.56, 0.60]	0.51 ± 0.09	0.44 ± 0.20
$IgG4_F$	140 ± 5.81	0.62 ± 0.18	0.70 ± 0.26	0.71 ± 0.14	0.19 ± 0.16	0.49 ± 0.08	[0.47, 0.51]	0.74 ± 0.30	0.21 ± 0.27
$C1q_F$	89.2 ± 12.0	0.70 ± 0.08	0.76 ± 0.083	0.98 ± 0.04	0.42 ± 0.098	0.82 ± 0.06	[0.80, 0.84]	0.63 ± 0.11	0.94 ± 0.11

Post-Dose 4									
	AIC (Mean ± SD)	Accuracy (Mean ± SD)	F1 (Mean ± SD)	PPV (Mean ± SD)	NPV (Mean ± SD)	AUC (Mean ± SD)	95%CI (AUC)	Sensitivity (Mean ± SD)	Specificity (Mean ± SD)
EU_F	82.1 ± 8.67	0.68 ± 0.09	0.73 ± 0.10	0.83 ± 0.09	0.49 ± 0.17	0.81 ± 0.08	[0.79, 0.83]	0.67 ± 0.14	0.67 ± 0.17
EU_{TOTAL}	76.1 ± 7.96	0.72 ± 0.08	0.77 ± 0.09	0.88 ± 0.08	0.53 ± 0.15	0.84 ± 0.08	[0.82, 0.86]	0.70 ± 0.12	0.78 ± 0.17
$IgG1_F$	90.9 ± 7.79	0.73 ± 0.09	0.80 ± 0.07	0.80 ± 0.10	0.58 ± 0.18	0.74 ± 0.09	[0.72, 0.77]	0.81 ± 0.11	0.55 ± 0.21
$IgG3_F$	104 ± 6.15	0.51 ± 0.07	0.59 ± 0.10	0.70 ± 0.10	0.31 ± 0.12	0.59 ± 0.10	[0.56, 0.62]	0.51 ± 0.11	0.50 ± 0.18
$IgG4_F$	107 ± 5.36	0.46 ± 0.11	0.53 ± 0.16	0.64 ± 0.11	0.24 ± 0.12	0.58 ± 0.08	[0.55, 0.60]	0.50 ± 0.21	0.39 ± 0.23
$C1q_F$	71.3 ± 5.34	0.63 ± 0.08	0.66 ± 0.10	0.90 ± 0.09	0.45 ± 0.11	0.74 ± 0.07	[0.72, 0.77]	0.53 ± 0.12	0.87 ± 0.13

Univariate analysis for %EU/%OD for EU_F, $IgG1_F$, $IgG3_F$, $IgG4_F$, $C1q_F$, and Pfs230D1 titers (EU_{TOTAL}). Statistical results reported for akaike information criterion (AIC), accuracy, F1 score (F1), positive predictive value (PPV), negative predictive value (NPV), 95% CI of AUC, sensitivity and specificity.

Table S4: Pairwise logistic regression of two P230Compete assay results for EU, ΔEU or ΔOD to predict TRA≥80%.

Post-Dose 3							
Metric	AIC (Mean ± SD)	Accuracy (Mean ± SD)	F1 (Mean ± SD)	PPV (Mean ± SD)	NPV (Mean ± SD)	AUC (Mean ± SD)	95%CI (AUC)
$IgG1_F$+$IgG3_F$	94.8 ± 8.82	0.72 ± 0.08	0.79 ± 0.07	0.92 ± 0.05	0.45 ± 0.12	0.83 ± 0.07	[0.82, 0.85]
$IgG1_F$+$IgG4_F$	95.3 ± 9.24	0.72 ± 0.08	0.78 ± 0.08	0.95 ± 0.05	0.46 ± 0.13	0.81 ± 0.08	[0.79, 0.83]
$IgG1_F$+EU_F	94.9 ± 10.9	0.75 ± 0.07	0.81 ± 0.06	0.94 ± 0.06	0.49 ± 0.14	0.86 ± 0.07	[0.84, 0.88]
$IgG1_F$+EU_{TOTAL}	97.9 ± 9.37	0.73 ± 0.07	0.79 ± 0.07	0.95 ± 0.05	0.47 ± 0.12	0.85 ± 0.07	[0.83, 0.87]
$IgG3_F$+$IgG4_F$	138 ± 5.11	0.51 ± 0.12	0.60 ± 0.13	0.77 ± 0.09	0.22 ± 0.12	0.57 ± 0.08	[0.55, 0.60]
$IgG3_F$+EU_F	110 ± 10.0	0.71 ± 0.08	0.78 ± 0.07	0.90 ± 0.06	0.44 ± 0.12	0.80 ± 0.09	[0.78, 0.98]
$IgG3_F$+EU_{TOTAL}	119 ± 8.13	0.70 ± 0.07	0.78 ± 0.06	0.88 ± 0.07	0.41 ± 0.11	0.75 ± 0.10	[0.72, 0.78]
$IgG4_F$+EU_F	110 ± 10.7	0.73 ± 0.07	0.81 ± 0.06	0.90 ± 0.06	0.47 ± 0.17	0.79 ± 0.08	[0.77, 0.82]
$IgG4_F$+EU_{TOTAL}	122 ± 8.33	0.69 ± 0.07	0.77 ± 0.06	0.87 ± 0.06	0.42 ± 0.14	0.75 ± 0.09	[0.72, 0.78]
EU_F+EU_{TOTAL}	111 ± 10.3	0.73 ± 0.08	0.81 ± 0.06	0.90 ± 0.06	0.48 ± 0.16	0.80 ± 0.08	[0.77, 0.82]

Post-Dose 4							
Metric	AIC (Mean ± SD)	Accuracy (Mean ± SD)	F1 (Mean ± SD)	PPV (Mean ± SD)	NPV (Mean ± SD)	AUC (Mean ± SD)	95%CI (AUC)
$IgG1_F$+$IgG3_F$	86.3 ± 7.81	0.70 ± 0.08	0.76 ± 0.08	0.85 ± 0.09	0.52 ± 0.13	0.79 ± 0.09	[0.77, 0.82]
$IgG1_F$ +$IgG4_F$	88.3 ± 7.27	0.70 ± 0.09	0.76 ± 0.09	0.84 ± 0.10	0.53 ± 0.15	0.77 ± 0.09	[0.75, 0.80]
$IgG1_F$ + EU_F	72.9 ± 8.67	0.77 ± 0.08	0.82 ± 0.07	0.90 ± 0.07	0.60 ± 0.14	0.87 ± 0.05	[0.86, 0.89]
$IgG1_F$+EU_{TOTAL}	67.0 ± 8.12	0.76 ± 0.08	0.81 ± 0.06	0.90 ± 0.08	0.59 ± 0.15	0.88 ± 0.06	[0.87, 0.90]
$IgG3_F$+$IgG4_F$	105 ± 5.36	0.50 ± 0.08	0.54 ± 0.12	0.71 ± 0.11	0.33 ± 0.12	0.59 ± 0.07	[0.58, 0.61]
$IgG3_F$+EU_F	85.1 ± 9.06	0.69 ± 0.10	0.75 ± 0.10	0.83 ± 0.09	0.52 ± 0.18	0.79 ± 0.10	[0.76, 0.82]
$IgG3_F$+EU_{TOTAL}	78.8 ± 8.31	0.72 ± 0.08	0.77 ± 0.08	0.85 ± 0.08	0.53 ± 0.15	0.82 ± 0.08	[0.80, 0.84]
$IgG4_F$+EU_F	84.8 ± 9.12	0.70 ± 0.10	0.76 ± 0.10	0.82 ± 0.08	0.53 ± 0.20	0.79 ± 0.08	[0.76, 0.81]
$IgG4_F$+EU_{TOTAL}	76.8 ± 8.19	0.72 ± 0.07	0.78 ± 0.07	0.85 ± 0.07	0.53 ± 0.16	0.82 ± 0.09	[0.80, 0.85]
EU_F+EU_{TOTAL}	76.7 ± 8.63	0.69 ± 0.08	0.75 ± 0.08	0.85 ± 0.07	0.50 ± 0.15	0.82 ± 0.08	[0.80, 0.84]

Combinations of two P230Compete assays to predict TRA≥80% including results for EU_F (analyzed as log-transformed ΔEU), $IgG1_F$, $IgG3_F$, and $IgG4_F$ (analyzed as ΔOD) and Pfs230D1 titers measured during the trial (EU_{TOTAL}, analyzed as log-transformed EU). $C1q_F$ results were excluded from analyses due to smaller datasets with limited sample availability.

Table S5: Multivariate logistic regression of three or more P230Compete assay results for ΔEU or ΔOD to predict TRA≥80%.

	AIC (Mean ± SD)	Accuracy (Mean ± SD)	F1 (Mean ± SD)	PPV (Mean ± SD)	NPV (Mean ± SD)	AUC (Mean ± SD)	Optimal combination of P230Compete
Post Dose 3	92.8 ± 9.23	0.75 ± 0.07	0.81 ± 0.07	0.92 ± 0.05	0.48 ± 0.13	0.85 ± 0.07	EU_F, $IgG1_F$, $IgG3_F$
Post Dose 4	68.0 ± 8.10	0.76 ± 0.07	0.81 ± 0.06	0.89 ± 0.08	0.58 ± 0.15	0.87 ± 0.06	EU_{TOTAL}, $IgG1_F$, $IgG3_F$

Datasets included in the multivariate models were EU_F (ΔEU, log-transformed) and EU_{TOTAL} (EU, log-transformed), as well as $IgG1_F$, $IgG3_F$, and $IgG4_F$ (ΔOD), age and sex with the optimal multivariate model reported post-dose 3 and 4. $C1q_F$ results were excluded from multivariate analyses.

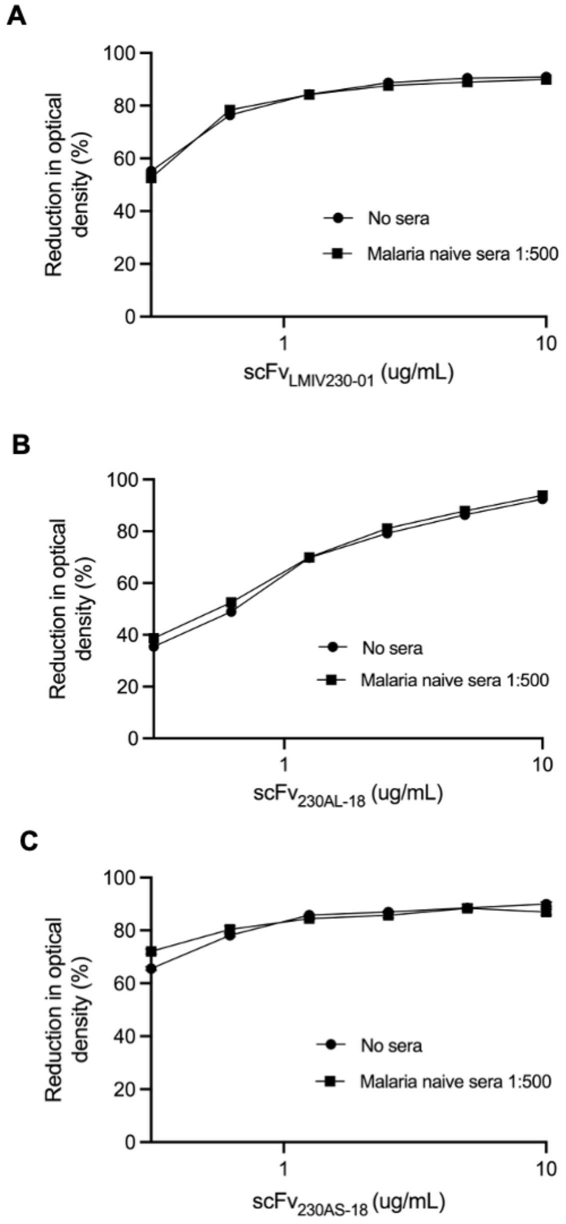

Fig. S1. Human serum does not impact the blocking function of scFv.
Competition experiments were performed with three single chain variable fragments (scFvs) with corresponding cognate human monoclonal antibody (hmAb) (A) LMIV230-01 (N=1), (B) 230AL-18 (N=3), and (C) 230AS-18 (N=1). The 3 scFvs were tested in a two-fold dilution series of 0-10 µg/mL (x-axis) with a constant hmAb concentration of 0.1 µg/mL in the presence (square) or absence (circle) of human malaria naïve sera. Y-axis is displacement of hmAb as percent reduction (%OD) relative to no scFv.

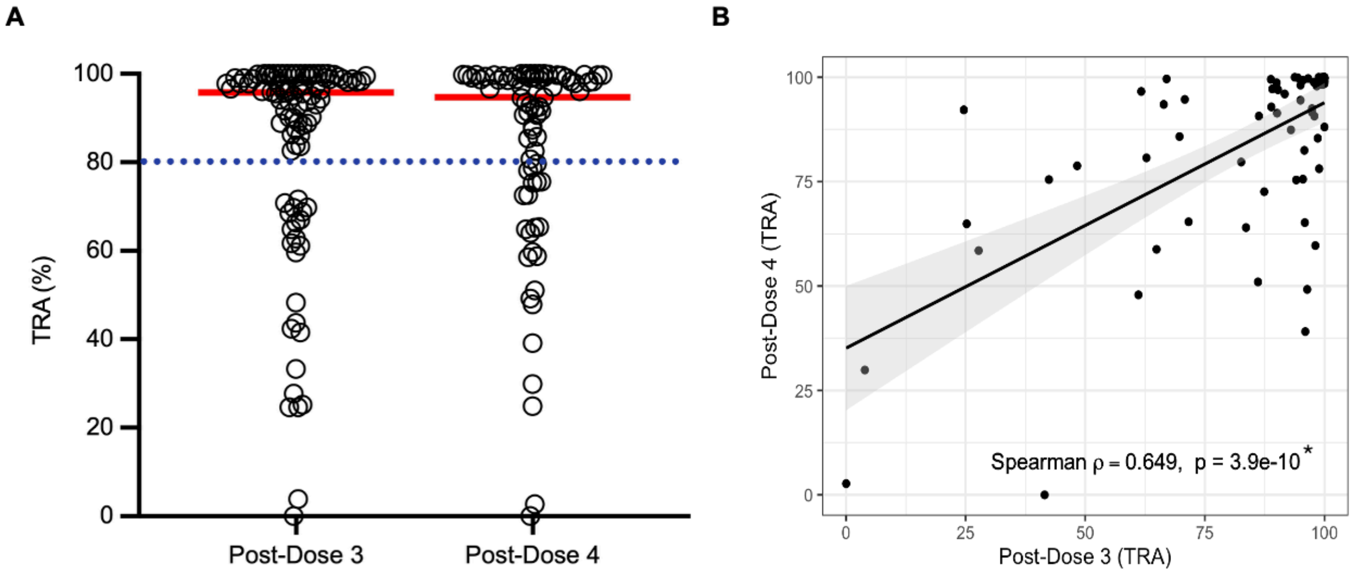

Fig. S2. TRA datasets used in P230Compete prediction analyses.
(A) Distribution of transmission reducing activity (TRA) values as measured by standard membrane feeding assay (SMFA) during the trial as reported in [14]. Black hollow circle indicated individual participant TRA value post-dose 3 (N = 94) and post-dose 4 (N = 77); red bars denote medians, and blue dashed line indicates TRA≥80% threshold. (B) Scatter plot shows the relationship between TRA values post-dose 3 and post-dose 4. Linear regression line fitted using least squares (black line) with 95% confidence intervals (gray shading) calculated as the fitted mean response ± 1.96 times the standard error (SE), assuming normally distributed residuals. * p-value calculated by Spearman rank correlation.

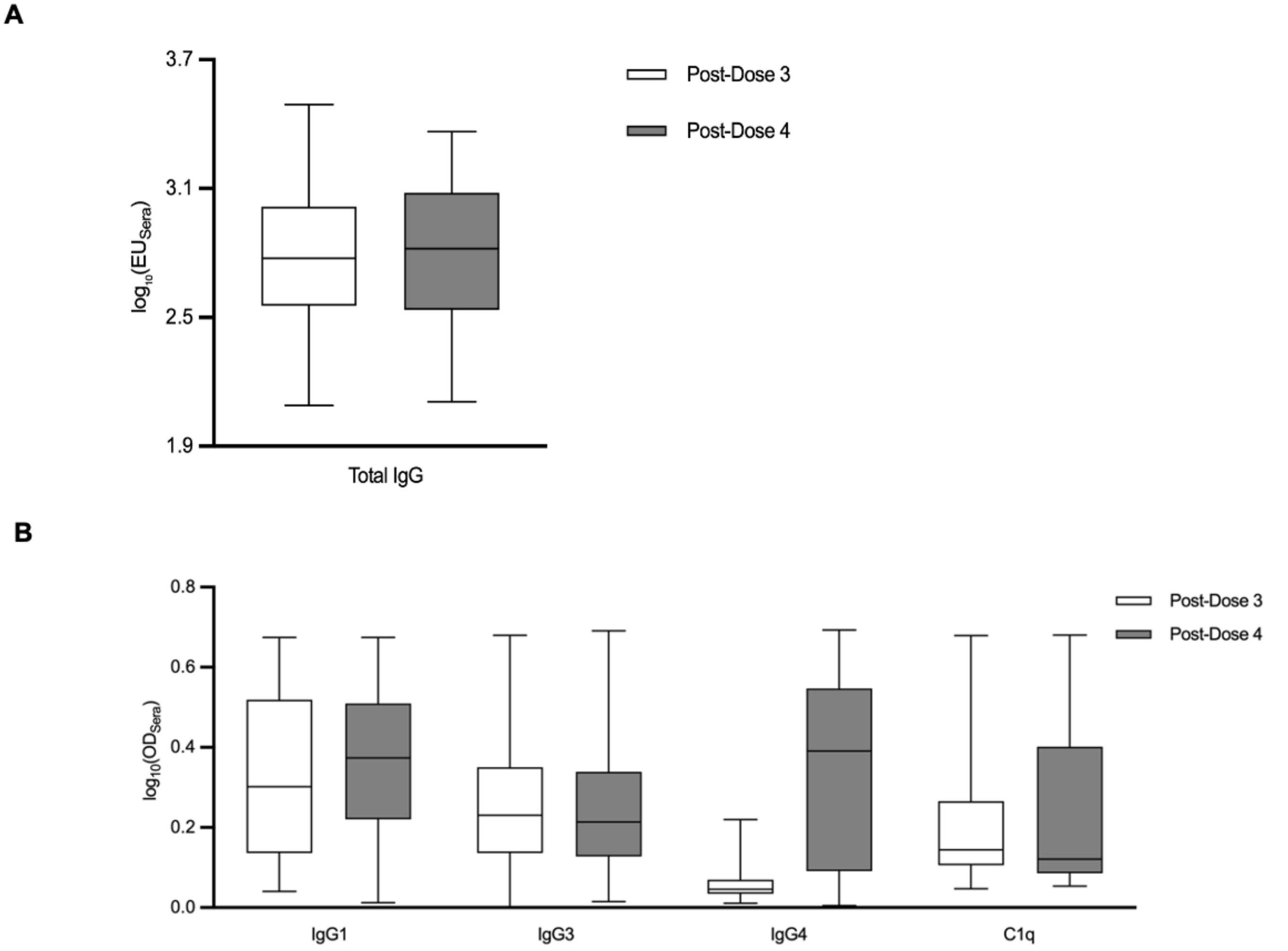

Fig. S3. Distribution of $\mathrm{EU}_{\mathrm{Sera}}$ and $\mathrm{OD}_{\mathrm{Sera}}$ results across P230Compete assay panel post-dose three and four. Box-and-whisker plots depict (A) total IgG results measured without scFv competitors (referred to as $\mathrm{EU}_{\mathrm{Sera}}$, expressed as log-transformed ELISA units: EU values), post-dose 3 (clear) and post-dose 4 (gray). (B) Results for $\mathrm{OD}_{\mathrm{Sera}}$ values for IgG1, IgG3, IgG4, and C1q measured without scFv competitors (log-transformed) with box plot post-dose 3 (clear) and box plot post-dose 4 (gray). Box plots indicate IQR (25th-75th percentile), with the horizontal line (median). Whiskers extend to 1.5xIQR with no outliers beyond this range.

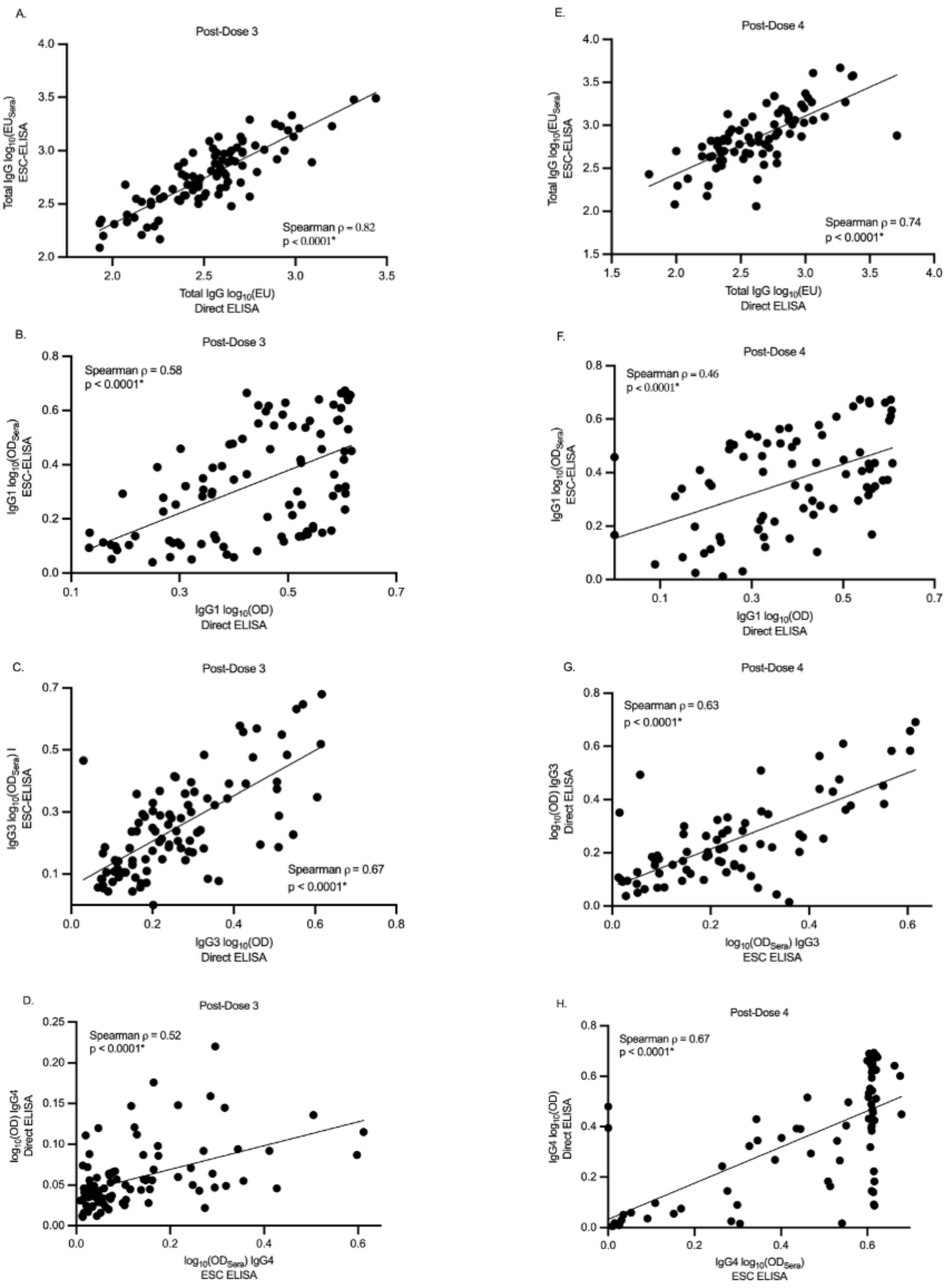

Fig. S4. Correlation between P230Compete OD_{Sera} results and Pfs230D1 titers measured by direct ELISA. P230Compete epitope-specific ELISA (ESC-ELISA) measurements with no scFv competitor (OD_{Sera}, x-axis) correlated to subclass Pfs230D1-specific titers measured by direct ELISA during the trial (y-axis) for total IgG and IgG subclasses. Each point represents the same serum sample measured for each assay platform; (A–D) Post-dose 3 (N = 94): (A) total IgG, (B) IgG1, (C) IgG3, (D) IgG4. (E–H) Post-dose 4 (N = 77): (E) total IgG, (F) IgG1, (G) IgG3, (H) IgG4. * p-value calculated by Spearman rank correlation.

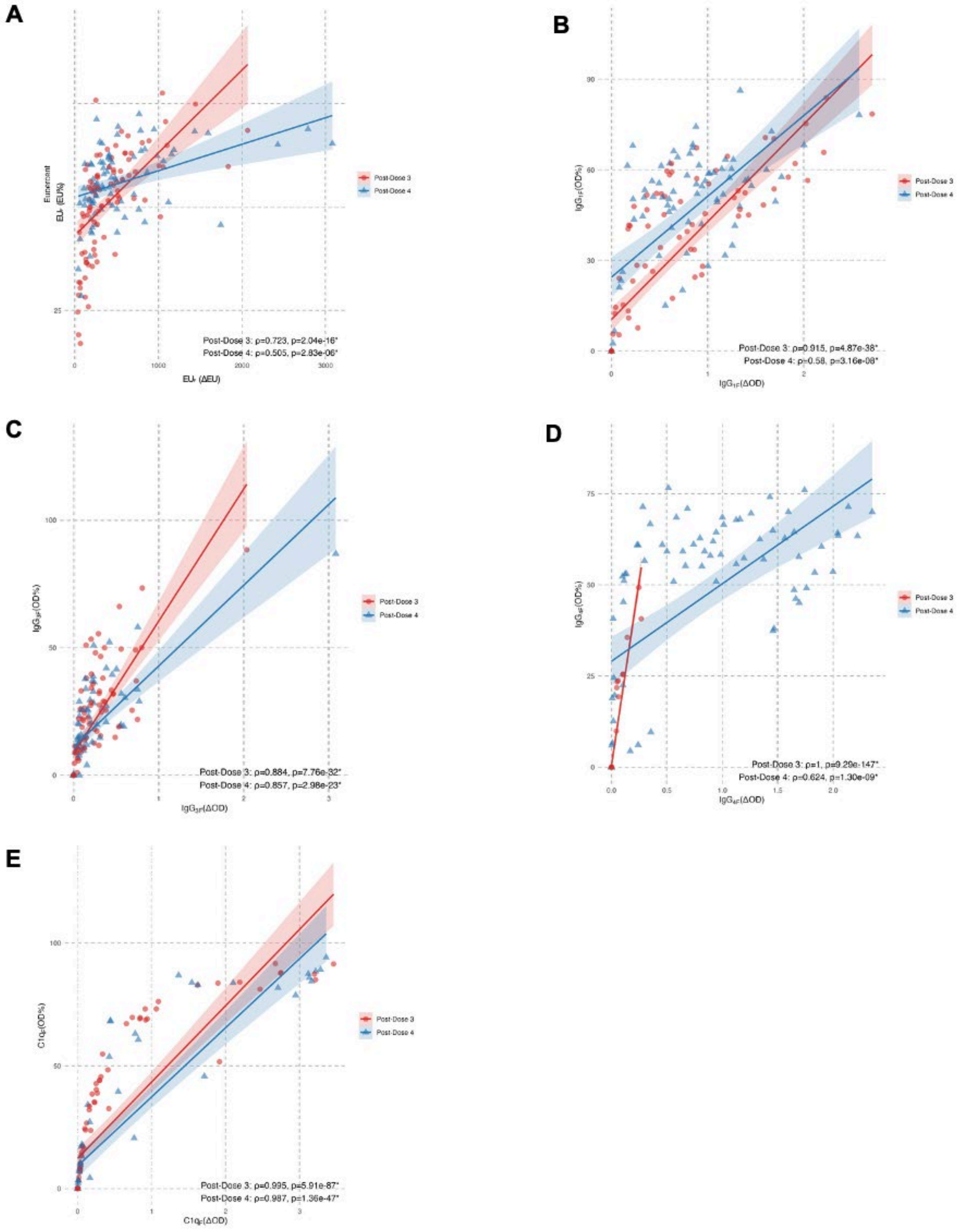

Fig. S5. Correlation between the two P230Compete measurements of percent reduction and delta reduction. Correlations of OD% vs ΔOD and EU% vs ΔEU, post-dose 3 (red circles; N = 94) and post-dose 4 (blue triangles; N = 77). (A) EU_F (EU values plotted); (B) $IgG1_F$, (C) $IgG3_F$, (D) $IgG4_F$, (E) $C1q_F$ (B-E: OD values plotted). Linear regression line fitted using least squares (red and blue lines) with 95% confidence intervals (red and blue shading) calculated as the fitted mean response ± 1.96 times the standard error (SE), assuming normally distributed residuals. * p-value calculated by Spearman rank correlation.

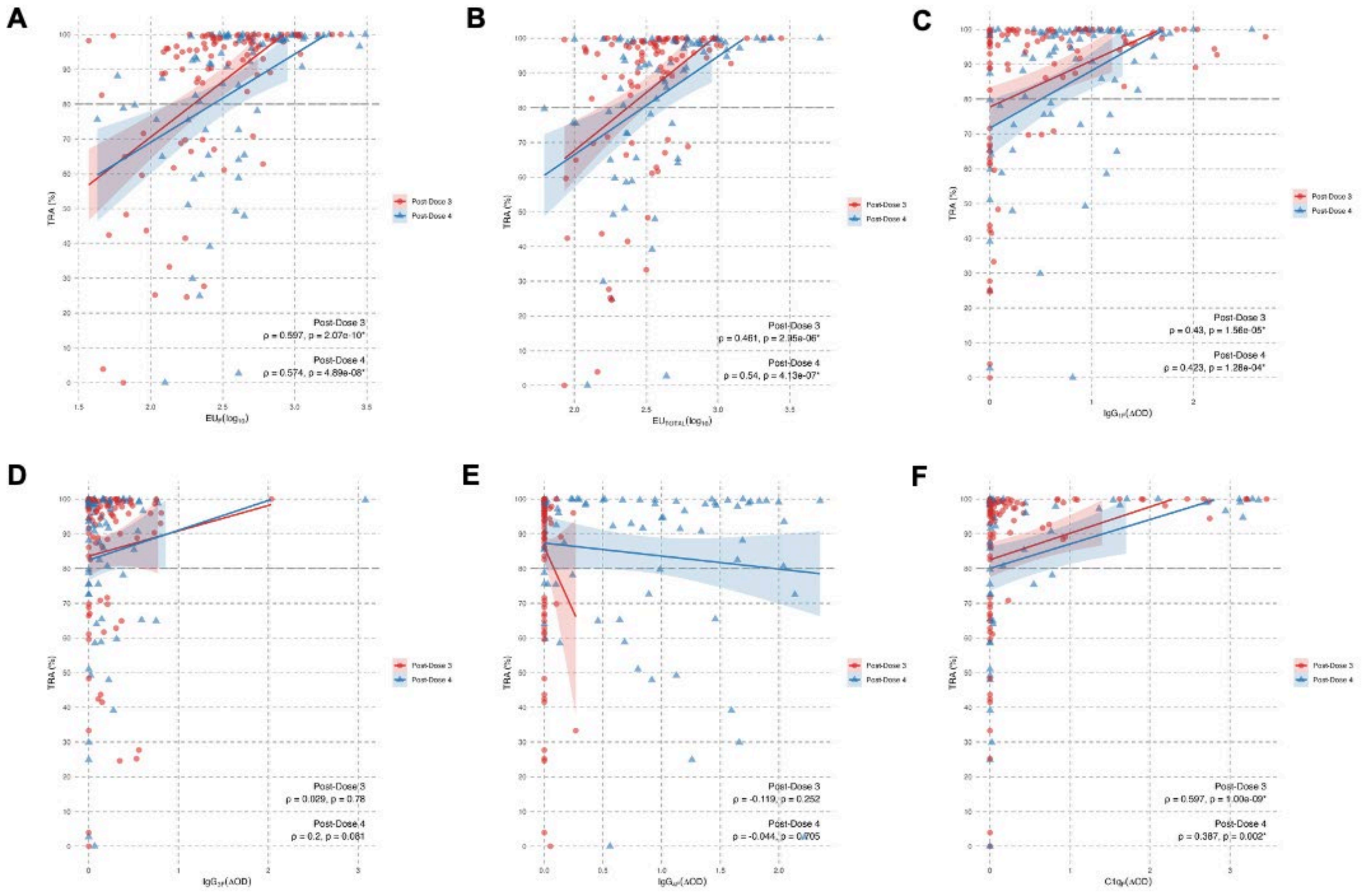

Fig S6. P230Compete results correlated to TRA.

Correlations of P230Compete levels vs. TRA (%), post-dose 3 (red circles; N = 94) and post-dose 4 (blue triangles; N = 77); gray dashed line represents the TRA≥80% threshold. (A) EU_F (ΔEU), (B) EU_{TOTAL} (EU), (C) $IgG1_F$ (ΔOD), (D) $IgG3_F$ (ΔOD), (E) $IgG4_F$ (ΔOD), and (F) $C1q_F$ (ΔOD). Linear regression line fitted using least squares (red and blue lines) with 95% confidence intervals (red and blue shading) calculated as the fitted mean response ± 1.96 times the standard error (SE), assuming normally distributed residuals. * p-value calculated by Spearman rank correlation.

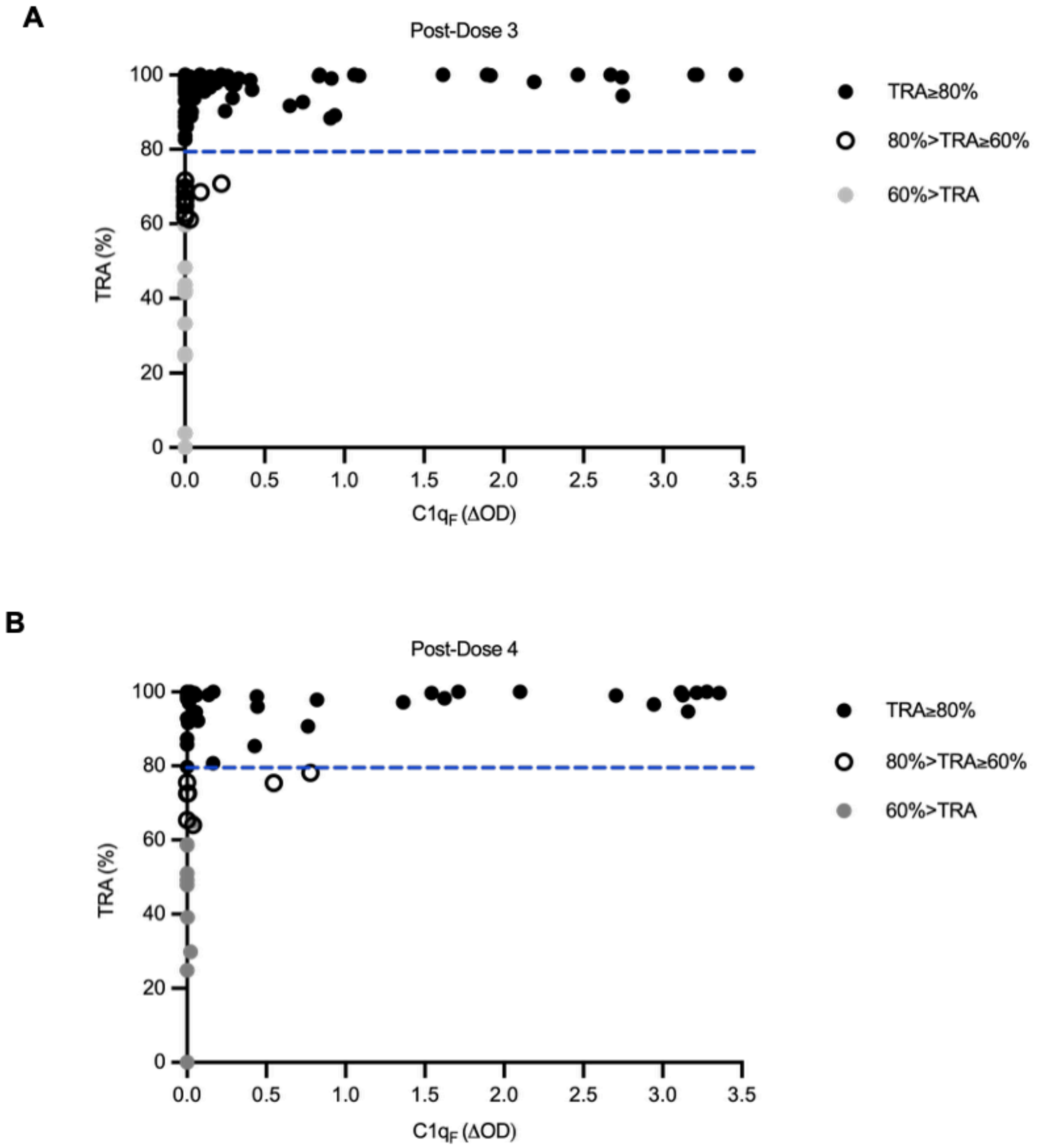

Fig. S7. C1q$_F$ levels differentiate high versus low TRA post-vaccination.
TRA (%) vs. C1q$_F$ (ΔOD): (A) post-dose 3 (N = 94), (B) post-dose 4 (N = 77). Samples grouped by TRA response high (TRA≥80%; black circles), intermediate (60%≤TRA<80%; black hollow circles), and low (TRA<60%; gray circles). Dashed blue line represents the TRA≥80% threshold.

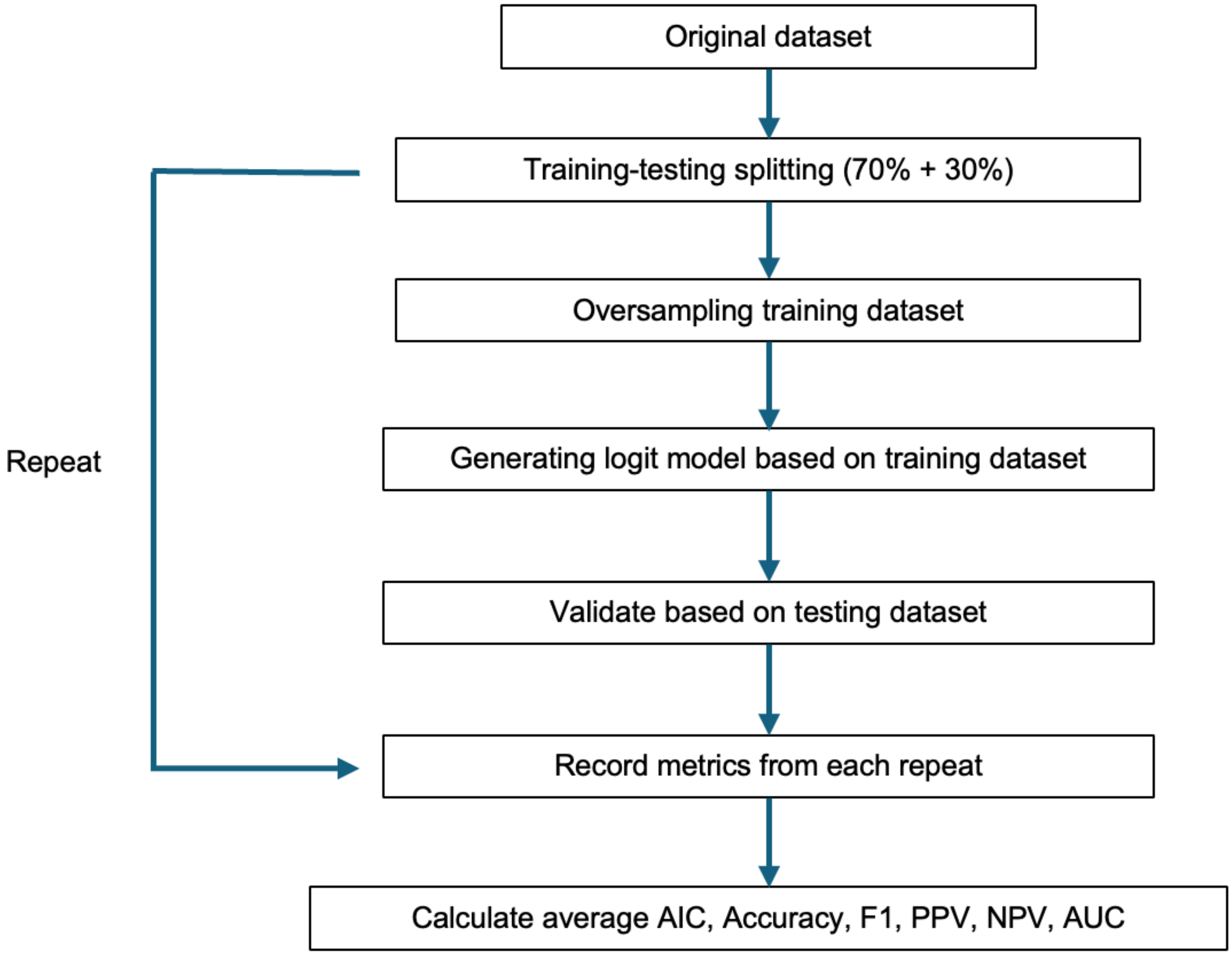

Fig. S8. Workflow for statistical prediction model construction and analysis.
Schematic of the statistical workflow used to construct and evaluate univariate, pairwise, and multivariate logistic regression models of P230Compete datasets to predict TRA≥80%. For each analysis, the dataset was randomly split into 70% training and 30% testing subsets, with oversampling applied to the training set to address class imbalance. Logistic regression models were trained on the re-sampled training dataset and evaluated on the testing dataset. This process was repeated multiple times to ensure robustness. Performance metrics were averaged across iterations including accuracy, F1 score, positive predictive value (PPV), negative predictive value (NPV), and AUC.

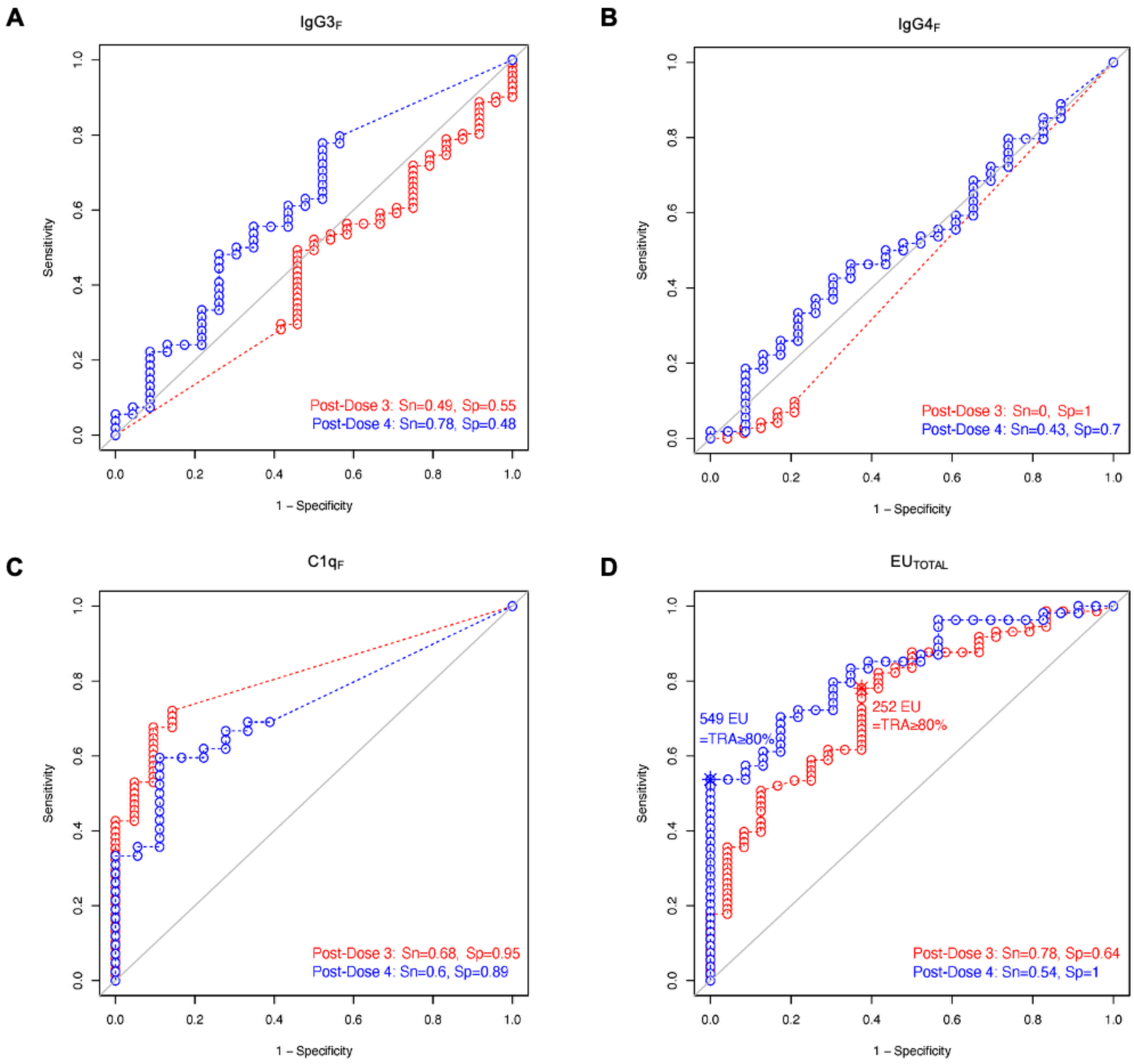

Fig. S9. P230Compete prediction of TRA by receiver operating characteristic curves.
ROC curves for: (A) $IgG3_F$, (B) $IgG4_F$, (C) $C1q_F$, (D) EU_{TOTAL} (for comparison), post-dose 3 (red; N = 94) and post-dose 4 (blue; N = 77). IgG isotyping and C1q binding as ΔOD and Pfs230D1 titers (EU_{TOTAL}) plotted as EU. Optimal cutoffs not calculated for $IgG3_F$, $IgG4_F$, or $C1q_F$ (AUC<0.80). EU_{TOTAL} cutoffs (in EU) marked on curve; gray diagonal line indicates AUC=0.5 (random classifier performance), Sn Sensitivity, Sp Specificity.

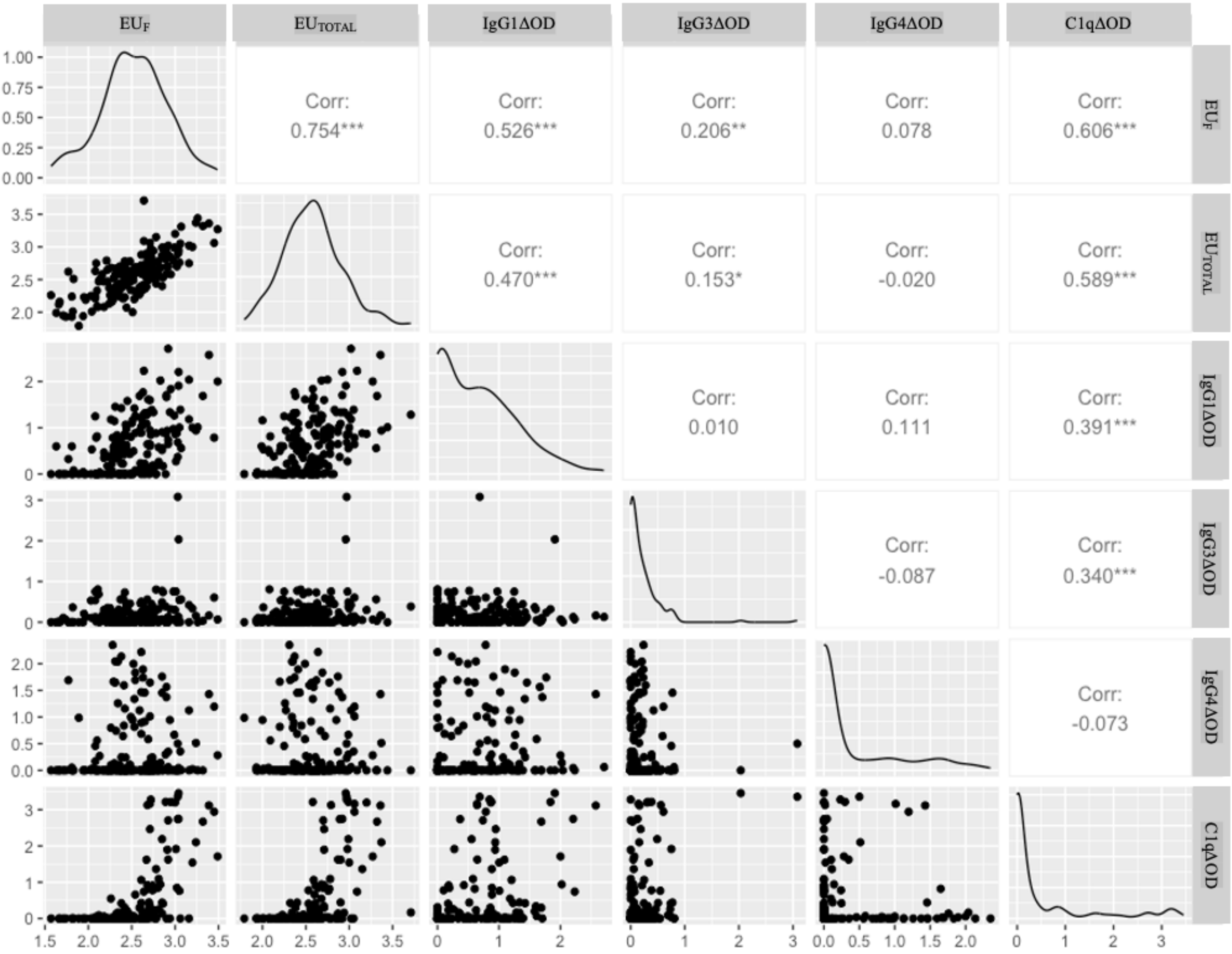

Fig. S10. Correlation analysis of P230Compete results to assess multicollinearity for multivariate modeling. Pairwise Spearman correlations across P230Compete assay panel datasets (EU_F expressed as ΔEU, EU_{TOTAL} expressed as EU, both datasets log-transformed). IgG and C1q datasets analyzed as ΔOD. Statistically significant Spearman rank correlations indicated by ***$p < 0.001$, **$p < 0.01$, *$p < 0.05$.
